# Triterpenoid Saponins From the Fruit of *Acanthopanax senticosus* (Rupr. & Maxim.) Harms

**DOI:** 10.3389/fchem.2022.825763

**Published:** 2022-02-21

**Authors:** Yan Liu, Peng Jiang, Mei-Ling Zhang, Juan Pan, Wei Guan, Xiao-Mao Li, Bing-You Yang, Hai-Xue Kuang

**Affiliations:** ^1^ Key Laboratory of Basic and Application Research of Beiyao (Heilongjiang University of Chinese Medicine), Ministry of Education, Harbin, China; ^2^ China Resources Double-Crane Pharmaceutical Co., Ltd., Peking, China

**Keywords:** triterpenoid saponins, *Acanthopanax senticosus* (Rupr. & Maxim.) Harms, fruit, cytotoxicity, neuroprotective

## Abstract

Five new oleanane-type triterpenoid saponins (**1–5**), together with 24 known saponins (**6–29**) were isolated from the fruit of *Acanthopanax senticosus*. Their structures were determined by extensive spectroscopic analysis, including 1D, 2D nuclear magnetic resonance (NMR), and high-resolution electrospray ionization mass spectrometry (HR-ESI-MS), in combination with chemical methods (acid hydrolysis). The neuroinflammation model was established by lipopolysaccharide (LPS)-induced BV2 microglia, and the neuroprotective effects of all compounds (**1–29**) were evaluated.

## Introduction

*Acanthopanax se* *nticosus* (Rupr. & Maxim.) Harms, commonly known as Ci Wu Jia or Siberian Ginseng, is a well-known traditional Chinese medicine widely distributed in the northeast of China. With high medicinal value, *A. senticosus* is popularly used as an “adaptogen” like Panax ginseng. Modern pharmacology study shows that this plant was used for antifatigue, anti-depression, anxiolytic, anti-irradiation, anticancer, anti-inflammatory, hypolipidemic, etc. (Huang et al., 2011; Li et al., 2016a), and these activities may be attributed to triterpenoid saponins. Insuperably, modern pharmacological studies have confirmed that *A. senticosus* fruits possess significant activities of antifatigue (Cong et al., 2010), antioxidant (Kim et al., 2015; Zhao et al., 2013), hypolipidemic (Yan et al., 2009), anti-obesity (Li et al., 2007; Saito et al., 2016), anti-inflammatory (Li et al., 2013), and so on. However, for the past few years, most of the phytochemical studies have been mainly focused on the root, stem, and leaves of *A. senticosus*, and limited researches have been investigated on its fruits (Ge et al., 2016; Huang et al., 2011; Li et al., 2016b; Li et al., 2017; Wang et al., 2012; Zhang et al., 2017).

**FIGURE 1:**
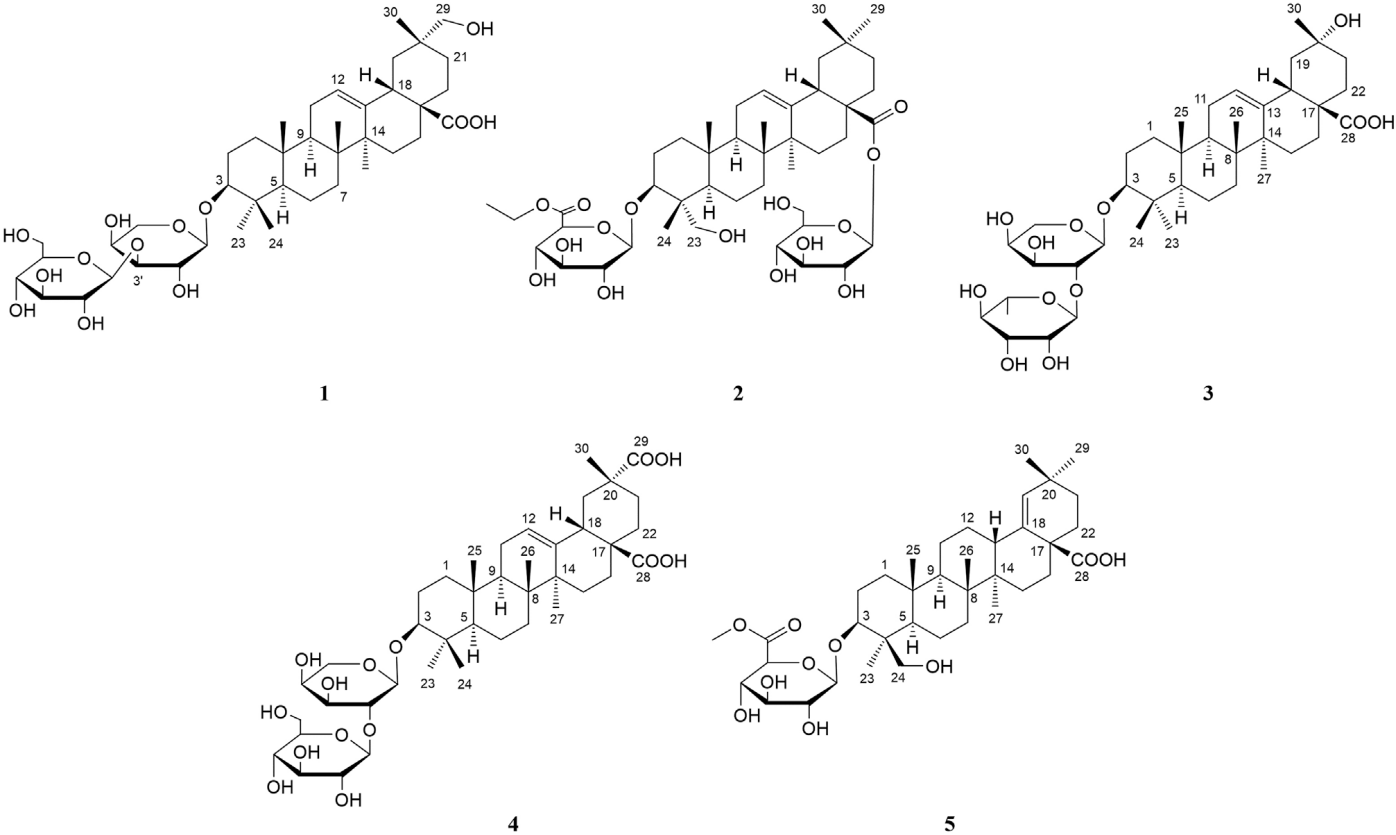
Chemical structures of compounds **1–5**.

In the present paper, we continue to further explore the active component triterpenoid saponins from the fruits of *A. senticosus.* The results found five previously undescribed triterpenoid saponins (**1–5**) (Figure 1), together with known 24 triterpenoid saponins (**6–29**). Their structures were elucidated mainly by spectroscopic methods including 1D and 2D nuclear magnetic resonance (NMR) experiments in combination with high-resolution electrospray ionization mass spectrometry (HR-ESI-MS) and by comparison of their physical and spectral data with literature. Meanwhile, their neuroprotective effects were evaluated by lipopolysaccharide (LPS)-induced BV2 microglia.

## Experimental Section

### General Experimental Procedures

The HR-ESI-MS data of the new triterpenoid saponins were obtained on a Thermo Orbitrap Fusion Lumos Tribrid Mass Spectrometer. The 1D and 2D NMR spectra were acquired on a Bruker DPX-600 spectrometer in Pyridine-d5 using TMS as internal standard. Preparative high-performance liquid chromatography (HPLC) (LC-20AR, Shimadzu) was performed on Waters Atlantis^®^ Prep T3 (5 μm, 10 × 250 mm column) with a RID-20 A detector, with flow rates of 3 ml/min. Optical rotation measurements were conducted on a JASCO P-2000 instrument. Gas chromatography-mass spectrometry (GC-MS) analysis was performed on an Agilent 7890A system with a DB-5 capillary column. Absorbance (OD) value was detected on a BioTek Epoch™2 Microplate Reader. The FT-IR data of the new triterpenoid saponins was performed on Thermo Scientific Nicolet iS10. Silica gel column chromatography (CC) and octadecyl silica (ODS) chromatography were used in the separation of extracts.

### Plant Material

The fruit of *A. senticosus* was collected in October 2018 from the Yichun, Heilongjiang Province. The plant was identified by the Professor Rui-Feng Fan of the Heilongjiang University of Chinese Medicine, and its voucher specimen (NO. 20190330) has been deposited at Heilongjiang University of Chinese Medicine.

### Extraction and Isolation

The dry fruits (20 kg) of *A. senticosus* were extracted with 70% EtOH three times, under reflux for 2 h each time to afford a crude extract (2,216 g). The crude extract was extracted with petroleum ether, EtOAc, and n-BuOH successively, and the corresponding extract was obtained after removing the solvent, namely, PE fraction (320.0 g), EtOAc fraction (470.0 g), and n-BuOH fraction (510.0 g). The ethyl acetate layer (360.0 g) was chromatographed on a silica gel column (200–300 mesh) eluted successively with CH_2_Cl_2_/MeOH (100:1–0:1) to obtain nine fractions. Fr. VI was separated on a silica gel column (200–300 mesh) eluted successively with CH_2_Cl_2_/MeOH (50:1–0:1) to obtain five fractions (Fr. VI 1–5). Fr. VI 4 was purified by ODS chromatography to afford 48 fractions. Fr. VI 4–(46) were purified by semi-preparative HPLC (MeOH/H_2_O 84%) to afford compounds **14** (5.3 mg), **7** (4.9 mg), **15** (5.0 mg), **8** (37.5 mg), **18** (9.7 mg), **16** (45.9 mg), **10** (7.1 mg), and **11** (5.4 mg). Fr. VII was separated on a silica gel column (200–300 mesh), using solvent system CH_2_Cl_2_/MeOH (30:1 to 0:1) to give five fractions (Fr. VII 1–7) based on TLC analysis. Fr. VII 5 was purified by ODS chromatography to afford sixty-three fractions. Fr. VII 5–(54) were purified by semi-preparative HPLC (MeOH/H_2_O 78%) to afford compounds **5** (5.3 mg) and **6** (3.6 mg). Fr. VIII was separated on a silica gel column (200–300 mesh, 1 kg), using solvent system CH_2_Cl_2_/MeOH (30:1 to 0:1) to give nine fractions (Fr. VIII 1–9) based on TLC analysis. Fr. VIII 5 was purified by ODS chromatography to afford fifty fractions. Fr. VIII 5–(47) were purified by semi-preparative HPLC (MeOH/H_2_O 84%) to afford compounds **13** (66.4 mg) and **9** (52.8 mg). Fr. VIII 6 was purified by ODS chromatography to afford forty-two fractions. Fr. VIII 6-(24) was purified by semi-preparative HPLC (MeOH/H_2_O 73%) to afford compound **3** (8.8 mg). Fr. VIII 6–(25) was purified by semi-preparative HPLC (MeOH/H_2_O 73%) to afford compound **12** (5.3 mg). Fr. VIII 6–(27) were purified by semi-preparative HPLC (MeOH/H_2_O 68%) to afford compounds **1** (26.0 mg), **4** (4.2 mg), **17** (147.1 mg), and **22** (26.4 mg). Fr. VIII 6–(29) was purified by semi-preparative HPLC (MeOH/H_2_O 73%) to afford compound **25** (42.0 mg). Fr. VIII 6–(29D) were purified by semi-preparative HPLC (MeOH/H_2_O 78%) to afford compound **19** (3.6 mg). Fr. VIII 6–(30) were purified by semi-preparative HPLC (MeOH/H_2_O 70%) to afford compound **23** (28.1 mg). Fr. VIII 6–(30C) were purified by semi-preparative HPLC (MeOH/H_2_O 80%) to afford compound **2** (8.7 mg). Fr. VIII 6–(32) were purified by semi-preparative HPLC (MeOH/H_2_O 80%) to afford compound **26** (5.4 mg). Fr. VIII 6–(33) were purified by semi-preparative HPLC (MeOH/H_2_O 81%) to afford compound **24** (9.1 mg). Fr. VIII 6–(34) were purified by semi-preparative HPLC (MeOH/H_2_O 82%) to afford compounds **28** (8.2 mg) and **27** (7.6 mg). Fr. VIII 6–(35) were purified by semi-preparative HPLC (MeOH/H_2_O 84%) to afford compounds **29** (2.8 mg), **20** (7.7 mg), and **21** (23.5 mg).

### Spectroscopic Data

Acasentrioid A (1): amorphous powder; ${\left[\text{α}\right]}_{\text{D}}^{24}=+14.7$ , (*c* = 0.15, MeOH); HR-ESI-MS *m/z*: 784.4845 [M + NH_4_]^+^ (calculated to be 784.4842 for C_41_H_70_NO_13_). The ^1^H (pyridine-*d*_5_, 600 MHz) and ^13^C NMR (pyridine-*d*_5_, 150 MHz) data are shown in Tables 1, 2.

**TABLE 1:** ^13^C NMR data (*δ*) for compounds **1–5** (150, MHz in pyridine-*d*_5_).

No	1	2	3	4	5
1	38.6	38.6	38.8	38.5	39.0
2	26.6	26.0	26.5	26.3	26.2
3	88.5	82.2	88.7	88.7	82.0
4	39.7	43.4	39.4	39.3	43.5
5	55.7	47.5	55.8	55.6	47.7
6	18.4	18.1	18.4	18.3	18.0
7	33.1	32.7	33.1	33.0	34.6
8	39.5	39.9	39.7	39.6	40.9
9	48.0	48.1	47.9	47.8	51.4
10	37.0	36.8	36.9	36.8	36.9
11	23.7	23.8	23.7	23.6	21.2
12	122.5	122.9	122.6	123.0	26.4
13	144.9	144.1	144.3	144.1	41.6
14	42.0	42.1	42.1	42.4	42.9
15	28.3	28.2	28.2	28.1	29.9
16	23.7	23.3	23.8	23.6	34.3
17	47.0	46.9	46.7	46.5	48.5
18	41.3	41.7	44.3	40.9	138.9
19	41.1	46.1	48.0	40.9	131.9
20	36.5	30.7	69.8	42.0	32.3
21	29.0	33.9	36.2	29.1	34.1
22	32.6	32.5	35.1	32.2	34.1
23	28.0	64.3	28.0	28.0	13.3
24	16.9	13.5	17.0	16.6	64.1
25	15.4	16.0	15.5	15.3	17.3
26	17.3	17.5	17.3	17.2	16.2
27	26.1	26.0	26.0	25.9	15.2
28	180.2	176.4	180.0	180.0	179.4
29	73.8	33.0	–	181.1	30.7
30	19.7	23.6	25.7	19.9	29.2
1′	107.3	106.4	104.9	104.6	106.4
2′	71.8	75.4	75.9	80.6	75.4
3′	84.1	77.9	73.9	73.3	77.8
4′	69.2	73.1	68.7	68.2	73.1
5′	67.0	77.4	64.8	64.8	77.2
6′	–	170.2	–	–	170.8
7′	–	61.1	–	–	51.9
8′	–	14.1	–	–	–
1″	106.3	95.6	101.7	105.7	–
2″	75.6	74.0	72.4	76.2	–
3″	78.3	78.9	72.6	78.0	–
4″	71.4	71.1	74.0	71.4	–
5″	78.6	79.2	69.8	78.0	–
6″	62.6	62.1	18.5	62.4	–

**TABLE 2:** ^1^H NMR data (*δ*) for compounds **1–5** (600, MHz in pyridine-*d*_5_).

No	1	2	3	4	5
1	0.94 *o*	0.95 *o*	0.90 *t* (13.7)	0.86 *t* (13.2)	0.93 *o*
	1.50 *o*	1.47 *dt* (3.5, 13.1)	1.47 *br s*	1.46 *o*	1.60 *d* (11.8)
2	1.88 *o*	1.98 *dd* (3.5, 13.1)	1.82 *o*	1.80 *t* (13.9)	1.99 *t* (12.9)
	2.14 *o*	2.22 *m*	2.07 *o*	2.05 *m*	2.24 *m*
3	3.33 *dd* (3.8, 11.5)	4.30 *dd* (4.3, 12.3)	3.25 *dd* (4.1, 11.5)	3.17 *dd* (4.2, 11.8)	4.32 *dd* (4.3, 12.1)
4	–	–	–	–	–
5	0.80 *d* (11.8)	1.64 *o*	0.75 *d* (11.3)	0.67 *d* (11.8)	1.60 *d* (11.8)
6	1.30 *o*	1.31 *br s*	1.28 *o*	1.22 *o*	1.30 *m*
	1.48 *o*	1.66 *o*	1.44 *br s*	1.42 *o*	1.67 *d* (12.1)
7	1.31 *o*	1.30 *br s*	1.28 *o*	1.22 *o*	1.41 *br s*
	1.47 *o*	1.58 *t* (11.9)	1.42 *br d* (14.2)	1.36 *m*	1.49 *o*
8	–	–	–	–	–
9	1.65 *t* (8.8)	1.72 *o*	1.60 *o*	1.57 *t* (8.6)	1.39 *br s*
10	–	–	–	–	–
11	1.91 *o*	1.90 *m*	1.87 *o*	1.87 *m*	1.17 *d* (11.1)
	–	–	–	–	1.46 *o*
12	5.51 *br s*	5.41 *t* (3.1)	5.53 *br s*	5.51 *t* (2.9)	1.28 *o*
	–	–	–	–	1.67 *d* (12.1)
13	–	–	–	–	2.69 *d* (11.9)
14	–	–	–	–	–
15	1.20 *o*	1.10 *o*	1.21 *br s*	1.18 *o*	1.24 *o*
	2.20 *o*	2.32 *td* (4.3, 13.6)	2.17 *t* (13.1)	2.15 *m*	1.99 *t* (12.9)
16	2.00 *br d* (10.7)	1.92 *br s*	2.03 *o*	2.00 *br d* (11.7)	1.41 *br s*
	2.23 *o*	2.03 *td* (3.7, 13.6)	2.26 *t* (13.1)	2.22 *t* (12.8)	2.51 *br d* (13.2)
17	–	–	–	–	–
18	3.42 *dd* (3.3, 13.7)	3.18 *dd* (4.0, 13.6)	3.35 *br d* (13.7)	3.41 *dd* (3.7, 13.7)	–
19	1.52 *o*	1.22 *m*	1.91 *o*	1.91 *o*	5.27 *s*
	2.18 *o*	1.70 *o*	2.44 *t* (13.7)	2.57 *t* (13.7)	–
20	–	–	–	–	–
21	1.40 *br d* (12.2)	1.07 *m*	1.82 *o*	1.80 *t* (13.9)	1.51 *o*
	1.85 *m*	1.33 *m*	2.03 *o*	2.30 *td* (4.3, 13.9)	1.74 *t* (9.3)
22	1.94 *br d* (13.6)	1.74 *o*	2.07 *o*	1.94 *o*	1.74 *t* (9.3)
	2.16 *o*	1.81 *td* (4.1, 13.8)	–	2.10 *m*	2.30 *m*
23	1.29 *s*	3.70 *d* (10.9)	1.17 *s*	1.18 *s*	0.92 *s*
	–	4.34 *d* (10.9)	–	–	–
24	0.96 *s*	0.93 *s*	1.06 *s*	1.00 *s*	3.70 *d* (11.0)
	–	–	–	–	4.34 *d* (11.0)
25	0.83 *s*	0.92 *s*	0.82 *s*	0.80 *s*	0.83 *s*
26	1.00 *s*	1.11 *s*	0.99 *s*	0.96 *s*	1.02 *s*
27	1.31 *s*	1.19 *s*	1.26 *s*	1.25 *s*	0.89 *s*
28	–	–	–	–	–
29	3.61 *s*	0.88 *s*	–	–	1.11 *s*
30	1.22 *s*	0.87 *s*	1.58 *s*	1.55 *s*	1.04 *s*
1′	4.74 *d* (7.3)	5.20 *d* (7.7)	4.91 *d* (5.1)	4.93 *d* (5.7)	5.22 *d* (7.7)
2′	4.58 *t* (7.3)	4.09 *t* (7.7)	4.57 *o*	4.58 *t* (5.7)	4.10 *t* (8.2)
3′	4.22 *o*	4.16 *m*	4.29 *o*	4.35 *o*	4.16 *t* (8.2)
4′	4.43 *br s*	4.46 *br s*	4.28 *o*	4.36 *br s*	4.45 *t* (9.6)
5′	3.75 *d* (11.9)	4.47 *o*	3.83 *d* (11.5)	3.78 *o*	4.47 *t* (9.6)
	4.20 *o*	–	4.32 *o*	4.28 *o*	–
6′	–	–	–	–	–
7′	–	4.23 *d* (7.1)	–	–	3.69 *s*
8′	–	1.14 *t* (7.1)	–	–	–
1″	5.39 *d* (7.7)	6.33 *d* (8.1)	6.16 *s*	5.17 *d* (7.8)	–
2″	4.03 *t* (7.7)	4.20 *d* (7.0)	4.75 *br s*	4.08 *t* (7.8)	–
3″	4.25 *t* (8.8)	4.27 *d* (8.8)	4.63 *dt* (3.1, 9.4)	4.18 *t* (8.9)	–
4″	4.24 *o*	4.37 *t* (9.2)	4.29 *o*	4.28 *o*	–
5″	3.98 *br s*	4.03 *m*	4.59 *o*	3.80 *o*	–
6″	4.39 *dd* (5.0, 11.7)	4.41 *dd* (4.3, 12.0)	1.63 *d* (5.9)	4.41 *dd* (4.2, 7.1)	–
	4.54 *br d* (10.8)	4.46 *dd* (2.2, 12.0)	–	–	–

Acasentrioid B (2): amorphous powder; ${\left[\text{α}\right]}_{\text{D}}^{24}=+7.5$ , (*c* = 0.32, MeOH); HR-ESI-MS *m/z*: 856.5043 [M + NH_4_]^+^ (calculated to be 856.5053 for C_44_H_74_NO_15_). The ^1^H (pyridine-*d*_5_, 600 MHz) and ^13^C NMR (pyridine-*d*_5_, 150 MHz) data are shown in Tables 1, 2.

Acasentrioid C (3): amorphous powder; ${\left[\text{α}\right]}_{\text{D}}^{24}=+2.1$ , (*c* = 0.28, MeOH); HR-ESI-MS *m/z*: 737.4490 [M + H]^+^ (calculated to be 737.4471 for C_40_H_65_O_12_). The ^1^H (pyridine-*d*_5_, 600 MHz) and ^13^C NMR (pyridine-*d*_5_, 150 MHz) data are shown in Tables 1, 2.

Acasentrioid D (4): amorphous powder; ${\left[\text{α}\right]}_{\text{D}}^{24}=+19.1$ , (*c* = 0.22, MeOH); HR-ESI-MS *m/z*: 798.4648 [M + NH_4_]^+^ (calculated to be 798.4634 for C_41_H_68_NO_14_). The ^1^H (pyridine-*d*_5_, 600 MHz) and ^13^C NMR (pyridine-*d*_5_, 150 MHz) data are shown in Tables 1, 2.

Acasentrioid E (5): amorphous powder; ${\left[\text{α}\right]}_{\text{D}}^{24}=-3.5$ , (*c* = 0.23, MeOH); HR-ESI-MS *m/z*: 663.4121 [M + H]^+^ (calculated to be 663.4103 for C_37_H_59_O_10_). The ^1^H (pyridine-*d*_5_, 600 MHz) and ^13^C NMR (pyridine-*d*_5_, 150 MHz) data are shown in Tables 1, 2.

### Hydrolysis of Compounds **1–5**

Monosaccharide was determined by GC (Teng et al., 2018). Compounds **1–5** (each 1.0 mg) were dissolved in 2 ml of 2 M HCl (dioxane/H_2_O, 1:1, *v*/*v*), and hydrolyzed at 90°C for 3 h. After removing dioxane in a vacuum, the solution was diluted with H_2_O and extracted with EtOAc (3 × 1 ml). The aqueous layer was evaporated to dryness. The dried residue was dissolved in pyridine (200 μl) and treated with *L*-cysteine methyl ester hydrochloride (2.0 mg). After stirring the mixture for 1 h at 60°C, 100 μl of N-trimethylsilylimidazole was added, and they were kept at 60°C for 1 h. The reaction mixture was suspended in 1.0 ml H_2_O and extracted with *n*-hexane (3 × 1.0 ml). The layer of *n*-hexane was directly analyzed by GC with a DM-5 column (30 m × 0.25 mm, 0.25 μm) with the elution of N_2_ as carrier gas. Other GC conditions are as follows: column temperature: 220–270°C with the rate of 3°C/min; injector and detector temperature: 250°C; split ratio: 10:1; and injection volume:1 μl. The configurations of *D*-glucose, *L*-arabinose, *D*-glucuronic acid, and *L*-rhamnose in compounds **1–5** were determined by comparison of their retention times with those of standard samples.

### Bioassay for Cytotoxicity Activities

In each well of a 96-well plate, 100 μl of logarithmic growth phase cells (density 1.5 × 105/ml) was inoculated and cultured at 37°C and 5% CO_2_ until the cells attached to the wall. A drug-containing medium (0, 50, 100, 200, 400, and 600 μM) was added to each well of the administration group, and an equal volume of medium was also added to the blank group, and they were incubate at 37°C and 5% CO_2_ for 24 h. After the culture, 10 μl of CCK-8 was added to each well and incubated for 1–4 h at 37°C and 5% CO_2_. The absorbance (OD) value of each well at 450 nm was detected with a microplate reader, repeating three times, and its IC_50_ value was calculated.

### Bioassay for NO Production Inhibitory Activities

The anti-neuroinflammatory effect of compounds **1**–**29** was evaluated by LPS-induced BV2 microglia reported previously (Luo et al., 2020). The BV2 microglia cells were plated into a 96-well plate. After adding LPS (1 μg/ml) to each well for 12 h, it was treated with or without compounds of various concentrations (0, 100, 200, 300, 400, and 600 μM) for 12 h. The NO production in the supernatant was measured by the Griess reaction. The absorbance at 570 nm was measured using a microplate reader. The NO concentration and the inhibitory rate were calculated through a calibration curve. Quercetin was used as the positive control. Experiments were repeated three times.

## Results and Discussion

### Structure Elucidation of Compounds

Compound **1** was obtained as an amorphous powder. The negative HR-ESI-MS showed a deprotonated molecular ion peak at *m/z* 784.4845 [M + NH_4_]^+^ (calculated for C_41_H_70_NO_13_, 784.4842), indicating its molecular formula of C_41_H_66_O_13_. The ^1^H NMR spectrum displayed characteristic resonances of an olean-12-ene skeleton, namely, six methyls [*δ*_H_ 0.83, 0.96, 1.00, 1.22, 1.29, and 1.31 (3H each, all s, H-25, 24, 26, 30, 23, and 27)], one oxygenated methylene [*δ*_H_ 3.61 (2H, s, H-29)], one oxygenated methine [*δ*_H_ 3.33 (1H, dd, *J* = 11.5, 3.8 Hz, H-3)], one olefin [*δ*_H_ 5.51 (1H, br. s, H-12)], and two anomeric proton signals [*δ*_H_ 4.74 (1H, d, *J* = 7.3 Hz, H-1′) and 5.39 (1H, d, *J* = 7.7 Hz, H-1″)] (see Tables 1, 2). Coupled with DEPT spectrum, the ^13^C NMR spectrum showed the presence of 41 signals, of which 30 signals were assigned to a triterpene of oleanane skeleton, containing one carboxyl group (*δ*_C_ 180.2), two olefinic carbons (*δ*_C_ 122.5 and 144.9), two anomeric carbons (*δ*_C_ 107.3 and 106.3), one downfield glycosylation-shifted oxygenated methine (*δ*_C_ 88.5), a hydroxymethyl carbon (*δ*_C_ 73.8), and six methyls (*δ*_C_ 15.4, 16.9, 17.3, 19.7, 26.1, and 28.0) (see Tables 1, 2). These observations implied that compound **1** might be an oleanane-type triterpenoid saponin.

**FIGURE 2:**
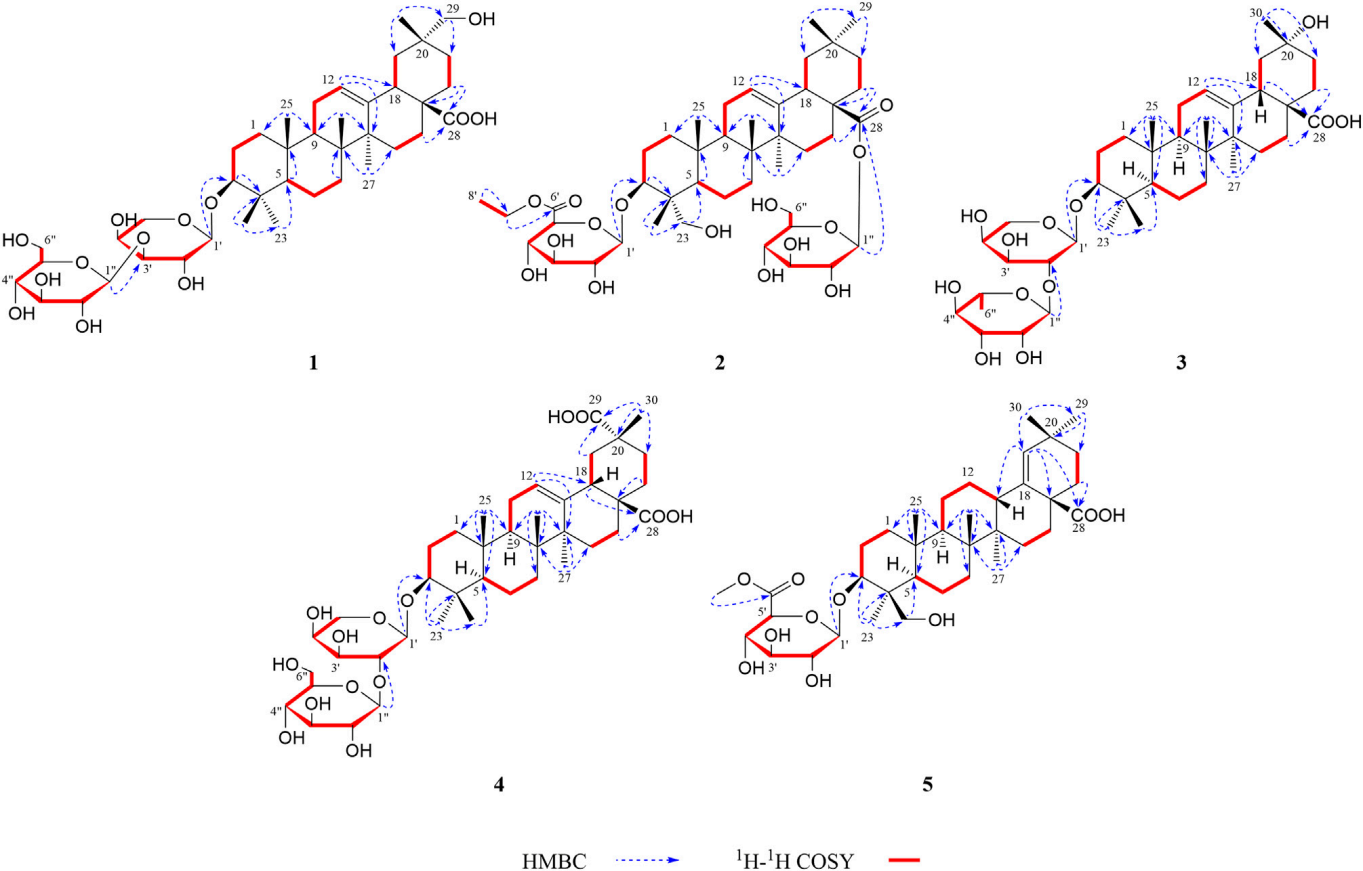
^1^H–^1^H COSY and key HMBC correlations of compounds **1–5**.

The HMBC cross-peaks of the anomeric proton H-1′ (*δ*_H_ 4.74)/C-3 showed the by ether bond location of the sugar chain at C-3. The HMBC connections of H_2_-22 (*δ*_H_ 2.16)/C-17, C-28 indicated a carboxy fragment attached at C-17 (Figure 2). Based on the above analysis, the structure of compound **1** was similar to compound **21**, and the major difference was the substituent C-29 is changed from methyl to oxymethylene in compound **1**, which was supported by the HMBC correlation of the anomeric protons of H_2_-29 (*δ*_H_ 3.61) with C-19, C-20, C-21, and C-30 (Figure 2). The β orientations of both pyranose sugars were deduced according to the large coupling constants of the anomeric protons (*J* = 7.3 Hz, H-1′; *J* = 7.7 Hz H-1″). To determine the absolute configuration of the arabinopyranose and glucopyranose, compound **1** was hydrolyzed by 2 mM HCl to obtain the sugar, and then, the trimethylsilyl thiazolidine derivatives of the sugar and standards, L-arabinose, and D-glucose were prepared. By comparing the retention times of these three trimethylsilyl thiazolidine derivatives obtained from GC, the absolute configuration of the arabinopyranose and glucopyranose in **1** was determined to be L and D, respectively.

**FIGURE 3:**
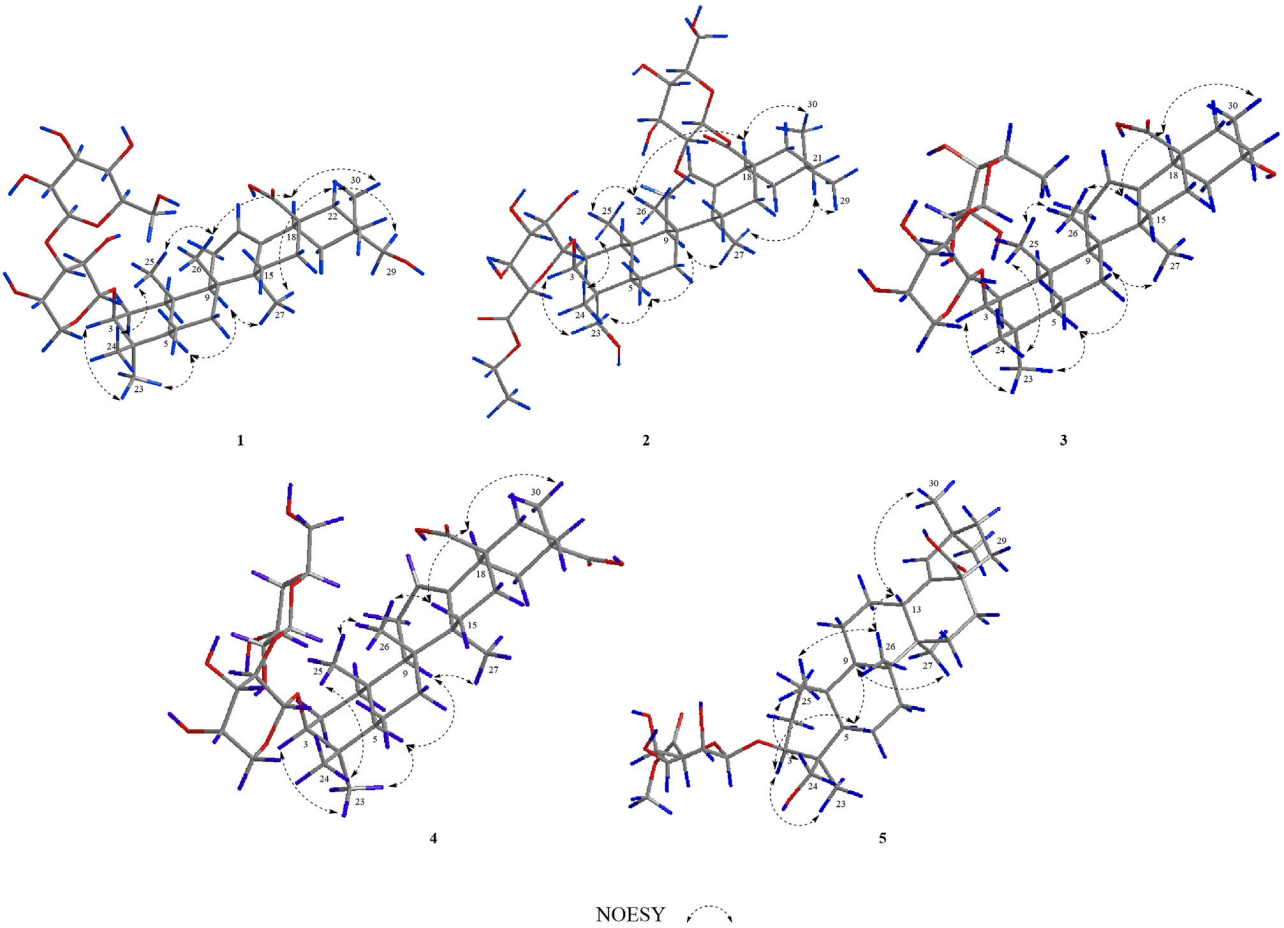
Key NOESY correlations of compounds **1–5**.

In the NOESY spectrum (Figure 3), the correlation peaks of H_3_-24/H_3_-25/H_3_-26/H-18/H_3_-30 suggested the β orientations of H_3_-24, H_3_-25, H_3_-26, H-18, and H_3_-30. Conversely, the correlation peaks of H-3/H_3_-23/H-5/H-9/H_3_-27/H-22*α* (*δ*_H_ 2.16)/H_2_-29 indicated that H-3, H-5, H-9, H_3_-23, H_3_-27, and H_2_-29 were α-oriented. Therefore, the structure of compound **1** was elucidated to be 3-*O*-*β*-glucopyranosyl-(1→3)-β-arabinopyranosyl-29-hydroxy-olean-12-en-28-oic acid, named acasentrioid A.

Compound **2** was isolated as an amorphous powder. Its molecular formula, C_44_H_70_O_15_, was determined by the negative HR-ESI-MS at *m/z* 856.5043 [M + NH_4_]^+^ (calculated for C_44_H_74_NO_15_, 856.5053). The ^1^H NMR spectrum displayed a skeleton characteristic similar to compound **1**, namely, six methyls [δ_H_ 0.87, 0.88, 0.92, 0.93, 1.11, and 1.19 (3H each, all s, H-30, 29, 25, 24, 26, and 27)], together with one methyl triplet at *δ*_H_ 1.14 (3H, t, *J* = 7.1 Hz, H-8′), one hydroxymethyl [*δ*_H_ 3.70 (1H, d, *J* = 10.9 Hz, H-23a), *δ*_H_ 4.34 (1H, d, *J* = 10.9 Hz, H-23b)], one oxygenated methine [*δ*_H_ 4.30 (1H, dd, *J* = 4.3, 12.3 Hz, H-3)], one olefin [*δ*_H_ 5.41 (1H, t, *J* = 3.1 Hz, H-12)], and two anomeric proton signals [*δ*_H_ 5.20 (1H, d, *J* = 7.7 Hz, GlcA-H-1′) and 6.33 (1H, d, *J* = 8.1 Hz, Glc-H-1″)] (see Tables 1, 2). There are 44 signals displayed in the ^13^C NMR spectrum, of which 30 signals corresponded to the triterpene of the oleanane skeleton. Combined with the DEPT spectrum, the ^13^C NMR spectrum showed resonances for one carboxyl group (*δ*_C_ 176.4), two olefinic carbons (*δ*_C_ 122.9 and 144.1), two anomeric carbons (*δ*_C_ 106.4 and 95.6), one downfield glycosylation-shifted oxygenated methine (*δ*_C_ 82.2), oxygenated methylene (*δ*_C_ 64.3), and seven methyls (*δ*_C_ 13.5, 14.1, 16.0, 17.5, 23.6, 26.0, and 33.0) (see Tables 1, 2). These observations implied that compound **2** might be as well an oleanane-type triterpenoid saponin.

The heteronuclear multiple bond coherence (HMBC) cross-peaks of the anomeric protons H-1′ (*δ*_H_ 5.20)/C-3 and H-1″ (*δ*_H_ 6.33)/C-28 show that sugar is attached to the ether bond of C-3 and C-28, respectively. The HMBC connections of H_2_-22 (*δ*_H_ 1.74, 1.81)/C-17, C-28 indicated a glycosylated carboxyl fragment attached at C-17 (Figure 2). Based on the above analysis, the structure of **2** was similar to ilexoside XLVIII (Amimoto et al., 1993), except for the ethyl group (*δ*_C_ 14.1 and 61.1) linked to C-6′ (*δ*_C_ 170.2) by ether bond in compound **2**, which was supported by the HMBC correlation of the ethyl protons H_3_-8′ and H-7′ with C-7′ and C-6′, respectively (Figure 2). The β orientations of both pyranose sugars were deduced according to the large coupling constants of the anomeric protons (*J* = 7.7 Hz, H-1′; *J* = 8.1 Hz, H-1″). The absolute configurations of both glucuronopyranosyl and glucopyranosyl were determined to be D by the same chemical methods and GC analysis as **1**.

In the NOESY spectrum (Figure 3), the correlation peaks of H_3_-24/H_3_-25/H_3_-26/H-18/H_3_-30 suggested the β orientations of H_3_-24, H_3_-25, H_3_-26, H-18, and H_3_-30. Conversely, the correlation peaks of H-3/H_2_-23/H-5/H-9/H-27/H-21*β* (*δ*_H_ 1.07)/H_3_-29 indicated that H-3, H-5, H-9, H_2_-23, H_3_-27, and H_3_-29 were α-oriented. Thus, the structure of compound **2** was defined as 3-*O*-*β*-*D*-6′-ethyl-glucuronopyranosyl-23-hydroxy-olean-12-en-28-*β*-*D*-glucopyranosyl, named acasentrioid B.

Compound **3** was also obtained as an amorphous powder with the molecular formula of C_40_H_64_O_12_ as indicated by the molecular ion peak *m/z* 737.4490 [M + H]^+^ (calculated 737.4471 for C_40_H_65_O_12_) in the HR-ESI-MS spectra. The ^1^H NMR spectra gave seven angular methyl signals at *δ*_H_ 0.82 (3H, s, H-25), 0.99 (3H, s, H-26), 1.06 (3H, s, H-24), 1.17 (3H, s, H-23), 1.26 (3H, s, H-27), 1.58 (3H, s, H-30), and 1.63 (3H, d, *J* = 5.9 Hz, Ara-H-6″); one oxygenated methine at *δ*_H_ 3.25 (1H, dd, *J* = 4.1, 11.5 Hz, H-3); one olefin at *δ*_H_ 5.53 (1H, br.s, H-12); and two anomeric proton signals at *δ*_H_ 4.91 (1H, d, *J* = 5.1 Hz, Ara-H-1′) and 6.16 (1H, s, Rha-H-1″) (see Tables 1, 2). Accordingly, the ^13^C NMR spectra also revealed seven methyl signals at *δ*_C_ 15.5 (C-25), 17.0 (C-24), 17.3 (C-26), 18.5 (C-6″), 26.0 (C-27), 28.0 (C-23), and 25.7 (C-30); an oxygen-substituted methine signal at *δ*_C_ 88.7 (C-3); oxygen-substituted quaternary carbon signal at *δ*_C_ 69.8 (C-20); two olefinic carbon signals at *δ*_C_ 122.6 (C-12) and 144.3 (C-13); and one carboxyl carbon signal at *δ*_C_ 180.0 (C-28), along with two anomeric carbon signals at *δ*_C_ 101.7 (C-1″) and 104.9 (C-1′) as determined by the HSQC and DEPT spectra. All these NMR data were characteristic resonances of olean-12-ene skeleton triterpenes. The NMR data of **3** resembled those of **17**, and the major difference was the substituent C-29 is changed from methyl to hydroxyl in compound **3**, which was supported by the chemical shift *δ*_C_ 69.8 (C-20) and the HMBC correlation of the anomeric protons of H_3_-30 (δ_H_ 1.58) with C-19, C-20, and C-21 (Figure 2). The HMBC connections of H_2_-22 (*δ*_H_ 2.07) and H-18 (*δ*_H_ 3.35)/C-28 indicated a carboxy fragment attached at C-17 (Figure 2). The absolute configurations of both arabinopyranose and rhamnopyranose were determined to be L by the same chemical methods and GC analysis as **1**. The coupling constant of the anomeric hydrogen *J* = 5.1 Hz (*δ*_H_ 4.91, d, H-1′) established the α-arabinopyranosyl linkage in **3**. In the NOESY spectrum (Figure 3), the correlation peaks of H_3_-24/H_3_-25/H_3_-26/H-15β/H-18/H_3_-30 indicated that the H_3_-24, H_3_-25, H_3_-26, H-18, and H_3_-30 were β-oriented. Conversely, the correlation peaks of H-3/H_3_-23/H-5/H-9/H_3_-27 suggested the α orientations of H-3, H-5, H-9, H_3_-23, H_3_-27, and OH-20. Thus, compound **3** was defined as 3*β-*[(*α*-L-rhamnopyranosyl-(1→2)-*α*-L-arabinopyranosyl)oxy]-20*α*,30-dihydroxy-norolean-12-en-28-oic acid, named acasentrioid C.

Compound **4** was obtained as an amorphous powder. Its molecular formula C_41_H_64_O_14_ was established by the HR-ESI-MS spectrum *m/z* 798.4648 [M + NH_4_]^+^ (calculated for C_41_H_68_NO_14_, 798.4634) and was supported by the ^13^C NMR spectroscopic data. The ^13^C NMR spectrum of **4** displayed 41 carbons, of which 30 were assigned to the aglycone part and the remaining 11 were assigned to two sugar units comprising one pentose and one hexose. The NMR spectra showed signals for six angular methyl at *δ*_H_ 0.80, 0.96, 1.00, 1.18, 1.25, and 1.55 (3H each, all s, H-25, 26, 24, 23, 27, and 30), and their corresponding carbons at *δ*_C_ 15.3 (C-25), 17.2 (C-26), 16.6 (C-24), 28.0 (C-23), 25.9 (C-27), and 19.9 (C-30); an olefinic group at *δ*_H_ 5.51 (1H, t, *J* = 2.9 Hz, H-12) and *δ*_C_ 123.0 (C-12) and 144.1 (C-13); one oxygenated methine at *δ*_H_ 3.17 (1H, dd, *J* = 4.2 and 11.8 Hz, H-3) and *δ*_C_ 88.7 (C-3); two anomeric proton signals at *δ*_H_ 4.93 (1H, d, *J* = 5.7 Hz, Ara-H-1′) and 5.17 (1H, d, *J* = 7.8 Hz, Glc-H-1″) and their corresponding carbons at *δ*_C_ 104.6 (C-1′) and 105.7 (C-1″); and two carboxy groups at *δ*_C_ 180.0 (C-28) and 181.1 (C-29), which were characteristic for the triterpenoid saponin with oleanane skeleton. Two-dimensional NMR data of **4** resembled those of **20**, and the major difference was the substituent C-29 is changed from methyl to carboxyl in compound **3**, which was supported by the chemical shift *δ*_C_ 181.1 (C-29) and the HMBC correlation of H-19 (*δ*_H_ 2.57)/C-29 and H_3_-30 (*δ*_H_ 1.55)/C-20, C-21, and C-29 (Figure 2). The absolute configurations of the arabinopyranose and glucopyranose were determined to be L and D, respectively, by the same chemical methods and GC analysis as **1**, and the coupling constant of anomeric protons *J* = 5.7 Hz (*δ*_H_ 4.93, d, H-1′) and *J* = 7.8 Hz (*δ*_H_ 5.17, d, H-1″) established the α-arabinopyranosyl and β-glucopyranosyl linkage in **4**. In the NOESY spectrum (Figure 3), the correlation peaks of H_3_-24/H_3_-25/H_3_-26/H-15*β*/H-18/H_3_-30 indicated that the H_3_-24, H_3_-25, H_3_-26, H-18, and H_3_-30 were β-oriented. Conversely, the correlation peaks of H-3/H_3_-23/H-5/H-9/H_3_-27 suggested the α orientations of H-3, H-5, H-9, H_3_-23, H_3_-27, and COOH-20. Thus, compound **4** was defined as 3*β*-[(O-*β*-D-glucopyranosyl-(1→2)-*α*-L-arabinopyranosyl)oxy]olean12-ene-28,29-dioic acid, named acasentrioid D.

Compound **5** was obtained as an amorphous powder. The HR-ESI-MS indicated a precise [M + H]^+^ ion at *m/z* 663.4121 (calculated for C_37_H_59_O_10_, 663.4103), indicating an empirical molecular formula of C_41_H_64_O_14_. In the ^1^H NMR spectrum, six quaternary methyl group protons at *δ*_H_ 0.83, 0.89, 0.92, 1.02, 1.04, and 1.11 (3H each, all s, H-25, 27, 23, 26, 30, and 29); a methoxy group proton at *δ*_H_ 3.69 (3H, s, H-7′); an olefinic proton at *δ*_H_ 5.27 (1H, s, H-19); an oxygenated methine proton at *δ*_H_ 4.32 (1H, dd, *J* = 4.3, 12.1 Hz, H-3), hydroxymethyl protons at *δ*_H_ 3.70 (1H, d, *J* = 11.0 Hz, H-24a) and *δ*_H_ 4.34 (1H, d, *J* = 11.0 Hz, H-24b); and an anomeric proton at *δ*_H_ 5.22 (1H, d, *J* = 7.7 Hz, GlcA-H-1′) along with six quaternary methyl group carbons at *δ*_C_ 17.3 (C-25), 15.2 (C-27), 13.3 (C-23), 16.2 (C-26), 29.2 (C-30), and 30.7 (C-29); methoxy group carbons at *δ*_C_ 51.9 (C-7′); an oxygen-bearing methine carbon at *δ*_C_ 82.0 (C-3); a set of olefinic carbons at *δ*_C_ 138.9 (C-18) and 131.9 (C-19); a hydroxymethyl carbon at *δ*_C_ 64.1 (C-24); a carboxyl carbon at *δ*_C_ 179.4 (C-28); an ester group carbon at *δ*_C_ 170.8 (C-6′); and an anomeric carbon at *δ*_C_ 106.4 (C-1′) in its 13C NMR suggested the aglycone belongs to oleanane-type triterpene (see Tables 1, 2). A detailed analysis of HSQC, HMBC, COSY, and NOESY spectra of **5** assisted the complete assignment of its ^1^H and ^13^C NMR data, which were similar to those of 3*β*,23-dihydroxyolean-18-en-28-oic acid (Cai and Geng, 2016). The only difference is the presence of glucuronopyranoside-6′-O-methyl ester at C-3 in **5**, while there is no glycoside at C-3 in 3*β*,23-dihydroxyolean-18-en-28-oic acid, which was further confirmed by the HMBC correlations of H-1′ with C-3 and of H_3_-7′ with C-6′ (Figure 2). The absolute configurations of the glucuronopyranosyl were determined to be D by the same chemical methods and GC analysis as **1**, and the coupling constant of anomeric proton *J* = 7.7 Hz (*δ*_H_ 5.22, d, H-1′) established the *β*-glucuronopyranoside in **5**. In the NOESY spectrum (Figure 3), the correlation peaks of H_2_-24/H_3_-25/H_3_-26/H-13/H_3_-30 indicated that the H_2_-24, H_3_-25, H_3_-26, H-13, and H_3_-30 were β-oriented. Conversely, the correlation peaks of H-3/H_3_-23/H-5/H-9/H_3_-27 suggested the α orientations of H-3, H-5, H-9, H_3_-23, and H_3_-27. Thus, compound **5** was defined as 3*β*,23-dihydroxyolean-18-en-28-oic acid 3-O-*β*-D-glucuronopyranoside-6′-O-methyl ester, named acasentrioid E.

The structures of known compounds **6–29** were determined as HN-saponin D1 (**6**) (Kizu et al., 1985), hederagenin glycosides 3-*O*-*α*-*L*-arabinopyranoside (**7**) (Grishkovets et al., 2005), oleanolic acid 3-*O*-*β*-*D*-glucuronopyranoside (**8**) (Li et al., 2012), HN-saponin K (**9**) (Kizu et al., 1985), 3-O-*β*-D-glucuronopyranosyl-3*β*,16*α*-dihydroxyolean-12-en-28-oic acid (**10**) (Ushijima et al., 2008), gypsogenin 3-O-glucuronide (**11**) (Bouguet-Bonnet et al., 2002), elatoside G (**12**) (Yoshikawa et al., 1995), hederagenin-3-*O*-*β*-*D*- glucuronopyranoside 6′-*O-*methyl ester (**13**) (Cao et al., 2011), tragopogonsaponin A methyl ester (**14**) (Warashina et al., 1991), 3-*O*-6′-*O*-methyl-*β*-*D*-glucuronopy-ranoside of gypsogenin (**15**) (Iwamoto et al., 1985), 3-*O*-*β*-*D*-(6′-*O*-methyl-glucuronopyranosyl) oleanolic acid (**16**) (Melek et al., 1996), 3-*O*-*α*-rhamnopyranose- (1→2)-*α*-arabinopyranosyl-29-hydroxy-olean-12-en-28-oic acid (**17**) (Shao et al., 1989), 3-O-[*α*-*L*-rhamnopyranosyl-(1→2)-*α*-*L*-arabinopyranosyl] oleanolic acid (**18**) (Nakanishi et al., 1993), HN-saponin F (**19**) (Mizui et al., 1988), saponin PE (**20**) (Zhong et al., 2001), oleanolic acid 3-O-*β*-D-glucopyranosyl (1→3)-*α*-L-arabinopyranoside (**21**) (Satoh et al., 1994), lucyoside F (**22**), lucyoside H (**23**) (Takemoto et al., 1984), 3-O-*β*-D-glucopyranosyl-(1→2)-*β*-D-glucopyranosyl oleanolic acid (**24**) (Reginatto et al., 2001), 3-*O*-*β*-*D*-glucuronopyranosyl methyl ester-28-*O*-*β*-*D*-glucopyranoside (**25**) (Li et al., 2007), oleanolic acid 3-O-*α*-L-rhamnopyranosyl(1→2)-*α*-L-arabinopyranosyl-28-O-*β*-D-glucopyranosyl ester (**26**) (Fan et al., 2013), paritriside E (**27**) (Wu et al., 2012), 3-O-*α*-arabinopyranosyl-(1→2)-*β*-glucopyranoside-30-norolean-12,20(29)-dien-28-oic acid (**28**) (Shao et al., 1989), and 3-[(O-*β*-D-glucopyranosyl-(1→3)-*α*-L-arabinopyranosyl)oxy]-30- noroleana-12,20(29)-dien-28-oic acid (**29**) (Jitsuno and Mimaki, 2010) by comparison with literature data (Supplementary Table S1). Compounds **7** and **20** were isolated from *A. senticosus* for the first time. Compounds **6**, **9**, **12**, **16**, **19**, **21**, **23**, and **24**, were isolated from *Acanthoganax* Miq. species for the first time. Compounds **10**, **11**, **14**, **15**, **22**, **26**, **27**, and **29** were isolated from the family Araliaceae for the first time.

### Bioactive Activity

The cytotoxicity of compounds **1–29** on BV2 microglia was determined by the CCK-8 assay, and the results are listed in Table 3. The neuroinflammation model was established by LPS-induced BV2 microglia, and the neuroprotective effect of compounds (**1**–**29**) *in vitro* was evaluated. Unfortunately, the results of the evaluation were not ideal. Compounds **5**, **10**, **12**, **13**, and **16** had moderate inhibitory effects on neuroinflammation, as indicated in Table 4, and other compounds had no anti-neuroinflammatory activity. Based on the existing results and analyzing its structure–activity relationship, we speculate in the structure of oleanane-type triterpene saponins; when the C-16 hydroxyl group is substituted or the structure contains only one methyl glucuronate, the compound has moderate anti-neuroinflammatory effects.

**TABLE 3:** Cytotoxic activities of compounds **1–29** on BV2 Cells (IC_50_, μM).

Compound	IC_50_ (μM)	Compound	IC_50_ (μM)
1	143.04 ± 10.89	16	102.31 ± 9.77
2	156.71 ± 12.67	17	234.75 ± 21.13
3	149.89 ± 13.55	18	203.62 ± 19.78
4	246.03 ± 21.16	19	465.70 ± 35.05
5	236.33 ± 20.21	20	136.24 ± 10.55
6	108.17 ± 8.24	21	306.45 ± 28.00
7	123.75 ± 10.56	22	145.61 ± 12.67
8	107.37 ± 9.59	23	131.47 ± 10.04
9	240.90 ± 18.55	24	188.55 ± 15.22
10	215.13 ± 20.24	25	276.32 ± 25.46
11	402.42 ± 39.54	26	160.31 ± 13.53
12	264.49 ± 22.89	27	132.56 ± 11.76
13	41.42 ± 3.93	28	565.45 ± 47.98
14	584.67 ± 45.59	29	126.57 ± 10.56
15	156.85 ± 11.00		

**TABLE 4:** Inhibitory effects of compounds **1–29** on NO in LPS-induced BV-2 Cells (*n* = 3, *x* ± *s*).

Compound	IC_50_ (μM)	Compound	IC_50_ (μM)
1	>100	16	92.55 ± 7.92
2	>100	17	>100
3	>100	18	>100
4	>100	19	>100
5	45.00 ± 3.89	20	>100
6	>100	21	>100
7	>100	22	>100
8	>100	23	>100
9	>100	24	>100
10	50.18 ± 4.72	25	>100
11	>100	26	>100
12	50.96 ± 5.05	27	>100
13	41.42 ± 3.93	28	>100
14	>100	29	>100
15	>100	Quercetin	10.50 ± 1.07

The IC_50_ > 100 μM was deemed inactive or meant ineffective.

## Conclusion

In summary, five previously undescribed oleanane-type triterpenoid saponins (**1–5**), together with twenty-four known saponins (**6–29**), were isolated from the fruit of *A. senticosus*. The structures of all compounds were elucidated by extensive spectroscopic analysis, including 1D, 2D NMR, and HR-ESI-MS, in combination with chemical methods (acid hydrolysis). The neuroinflammation model was established by LPS-induced BV2 microglia, and the neuroprotective effects of all compounds (**1–29**) were evaluated. Unfortunately, the results of the evaluation were not ideal. Compounds **5, 10, 12, 13,** and **16** had moderate inhibitory effects on neuroinflammation, while other compounds have no anti-neuroinflammatory activity.

## Data Availability

The original contributions presented in the study are included in the article/Supplementary Material; further inquiries can be directed to the corresponding authors.
