# Supplementary figures and images for: Triterpenoid Saponins From the Fruit of Acanthopanax senticosus (Rupr. & Maxim.) Harms
Source: Front Chem. 2022 Feb 21;10:825763. doi: 10.3389/fchem.2022.825763 (PMC8899614; doi:10.3389/fchem.2022.825763)

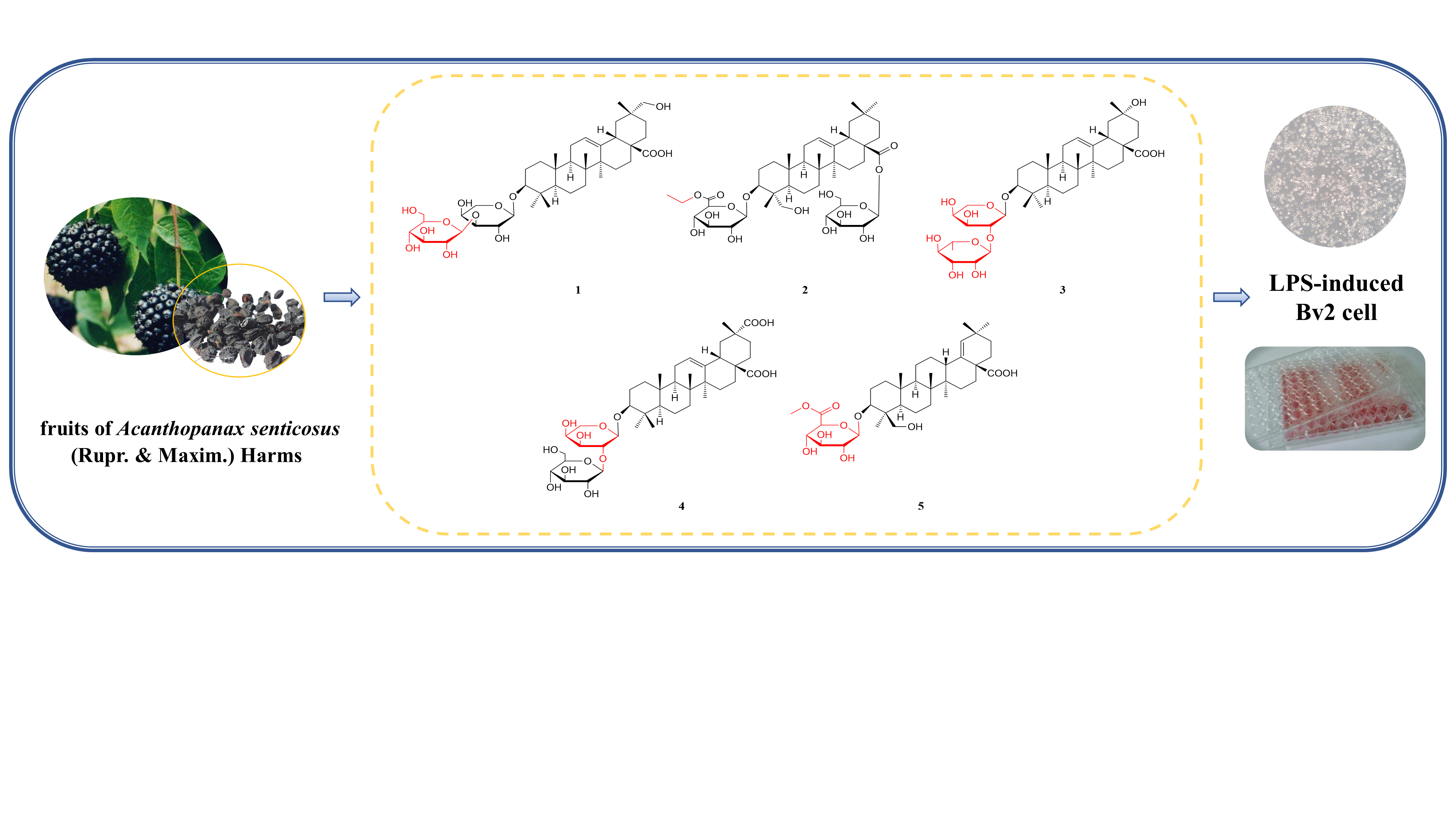

Supplement: Supplementary file 1 [file Image1.JPEG]
