# Supplementary material for: Triterpenoid Saponins From the Fruit of Acanthopanax senticosus (Rupr. & Maxim.) Harms
Source: Front Chem. 2022 Feb 21;10:825763. doi: 10.3389/fchem.2022.825763 (PMC8899614; doi:10.3389/fchem.2022.825763)
Supplement: Supplementary file 2 [file DataSheet1.pdf]

Supplementary Material

Supplementary Figures

Figure S1. ^{1}H NMR spectrum of Acasentrioid A (**1**) (600 MHz, pyridine-d5).
Figure S2. ^{13}C NMR spectrum of Acasentrioid A (**1**) (150 MHz, pyridine-d5).
Figure S3. DEPT spectrum of Acasentrioid A (**1**).
Figure S4. HSQC spectrum of Acasentrioid A (**1**).
Figure S5. ^{1}H-^{1}H COSY spectrum of Acasentrioid A (**1**).
Figure S6. HMBC spectrum of Acasentrioid A (**1**).
Figure S7. NOESY spectrum of Acasentrioid A (**1**).
Figure S8. HR-ESI-MS spectrum of Acasentrioid A (**1**).
Figure S9. CD spectrum of Acasentrioid A (**1**).
Figure S10. IR spectrum of Acasentrioid A (**1**).
Figure S11. ^{1}H NMR spectrum of Acasentrioid B (**2**) (600 MHz, pyridine-d5).
Figure S12. ^{13}C NMR spectrum of Acasentrioid B (**2**) (150 MHz, pyridine-d5).
Figure S13. DEPT spectrum of Acasentrioid B (**2**).
Figure S14. HSQC spectrum of Acasentrioid B (**2**).
Figure S15. ^{1}H-^{1}H COSY spectrum of Acasentrioid B (**2**).
Figure S16. HMBC spectrum of Acasentrioid B (**2**).
Figure S17. NOESY spectrum of Acasentrioid B (**2**).
Figure S18. HR-ESI-MS spectrum of Acasentrioid B (**2**).
Figure S19. CD spectrum of Acasentrioid B (**2**).
Figure S20. IR spectrum of Acasentrioid B (**2**).
Figure S21. ^{1}H NMR spectrum of Acasentrioid C (**3**) (600 MHz, pyridine-d5).
Figure S22. ^{13}C NMR spectrum of Acasentrioid C (**3**) (150 MHz, pyridine-d5).
Figure S23. DEPT spectrum of Acasentrioid C (**3**).
Figure S24. HSQC spectrum of Acasentrioid C (**3**).
Figure S25. ^{1}H-^{1}H COSY spectrum of Acasentrioid C (**3**).
Figure S26. HMBC spectrum of Acasentrioid C (**3**).
Figure S27. NOESY spectrum of Acasentrioid C (**3**).
Figure S28. HR-ESI-MS spectrum of Acasentrioid C (**3**).
Figure S29. CD spectrum of Acasentrioid C (**3**).
Figure S30. IR spectrum of Acasentrioid C (**3**).
Figure S31. ^{1}H NMR spectrum of Acasentrioid D (**4**) (600 MHz, pyridine-d5).
Figure S32. ^{13}C NMR spectrum of Acasentrioid D (**4**) (150 MHz, pyridine-d5). **Figure S33**. DEPT spectrum of Acasentrioid D (**4**).
Figure S34. HSQC spectrum of Acasentrioid D (**4**).
Figure S35. ^{1}H-^{1}H COSY spectrum of Acasentrioid D (**4**).
Figure S36. HMBC spectrum of Acasentrioid D (**4**).
Figure S37. NOESY spectrum of Acasentrioid D (**4**).
Figure S38. HR-ESI-MS spectrum of Acasentrioid D (**4**).
Figure S39. CD spectrum of Acasentrioid D (**4**).
Figure S40. IR spectrum of Acasentrioid D (**4**).
Figure S41. ^{1}H NMR spectrum of Acasentrioid E (**5**) (600 MHz, pyridine-d5).
Figure S42. ^{13}C NMR spectrum of Acasentrioid E (**5**) (150 MHz, pyridine-d5).

Figure S43. DEPT spectrum of Acasentrioid E (**5**).
Figure S44. HSQC spectrum of Acasentrioid E (**5**).
Figure S45. ^{1}H-^{1}H COSY spectrum of Acasentrioid E (**5**).
Figure S46. HMBC spectrum of Acasentrioid E (**5**).
Figure S47. NOESY spectrum of Acasentrioid E (**5**).
Figure S48. HR-ESI-MS spectrum of Acasentrioid E (**5**).
Figure S49. CD spectrum of Acasentrioid E (**5**).
Figure S50. IR spectrum of Acasentrioid E (**5**).
Figure S51. GC-MS analysis data of compound (**1**) hydrolysis
Figure S52. GC-MS analysis data of compound (**2**) hydrolysis
Figure S53. GC-MS analysis data of compound (**3**) hydrolysis
Figure S54. GC-MS analysis data of compound (**4**) hydrolysis
Figure S55. GC-MS analysis data of compound (**5**) hydrolysis
Figure S56. Structures of compounds **6-29**
Table S1. ^{13}C NMR data of **3-29**

Supplementary Figures

Figure S1. ^{1}H NMR spectrum of Acasentrioid A (**1**) (600 MHz, pyridine-d5).

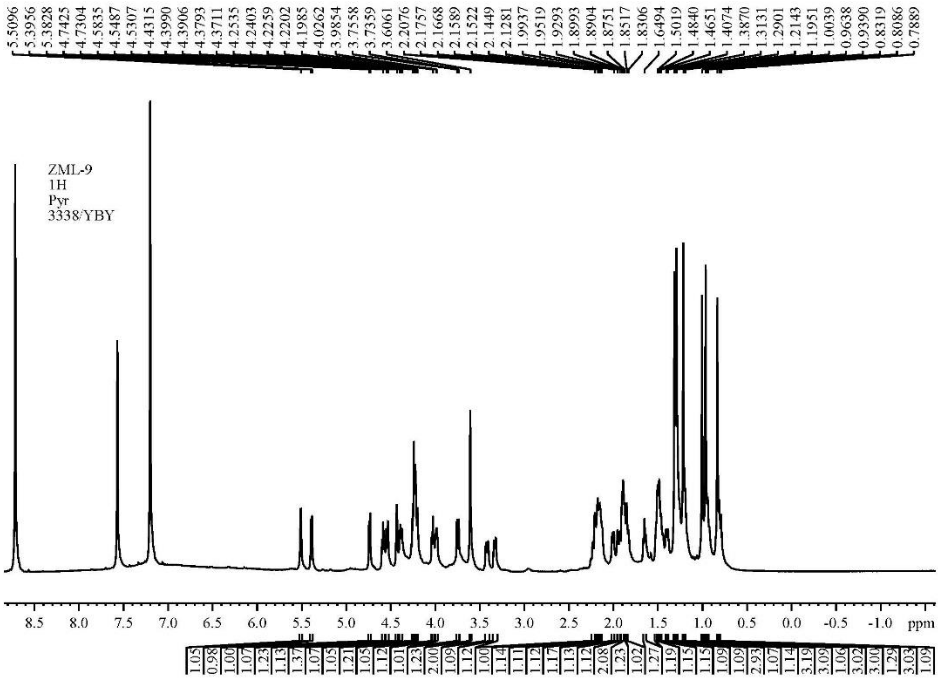

Figure S2. ^{13}C NMR spectrum of Acasentrioid A (**1**) (150 MHz, pyridine-d5).

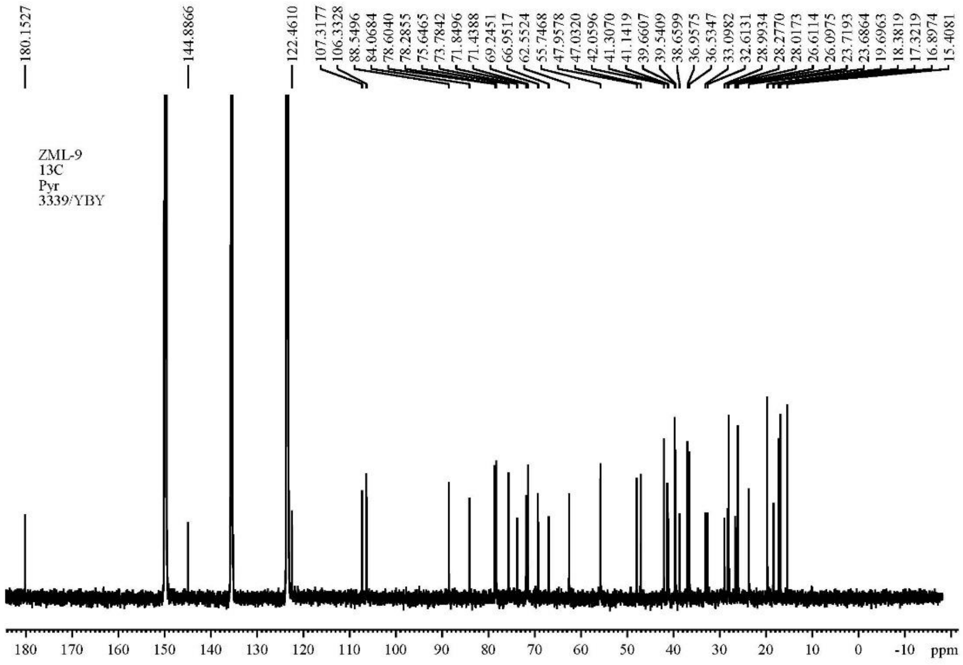

Figure S3. DEPT spectrum of Acasentrioid A (**1**).

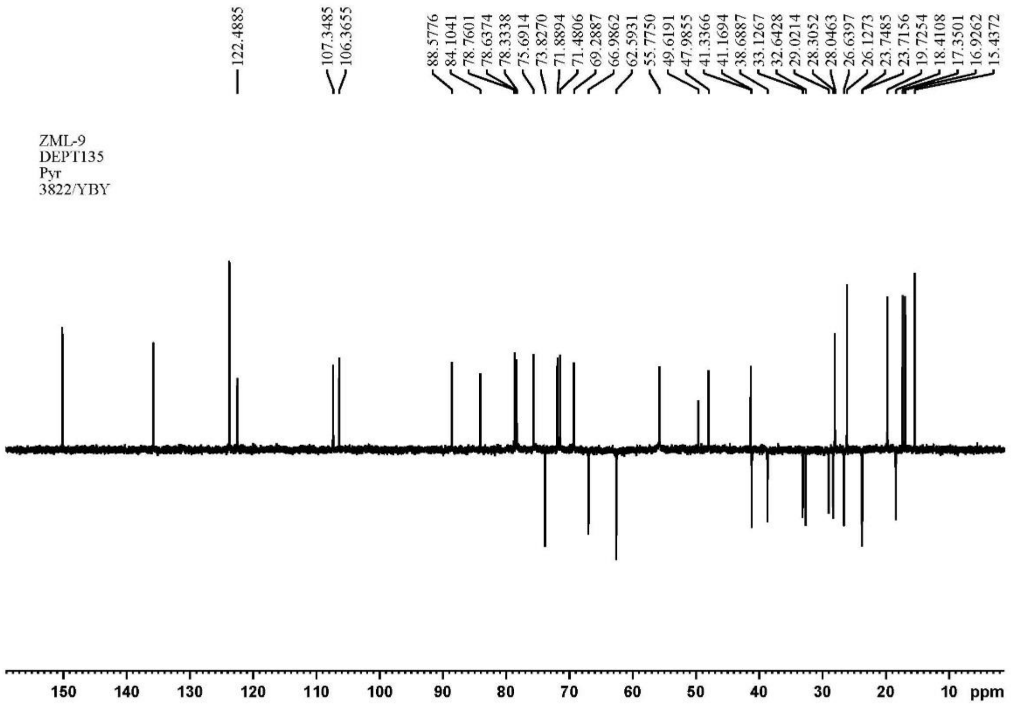

Figure S4. HSQC spectrum of Acasentrioid A (**1**).

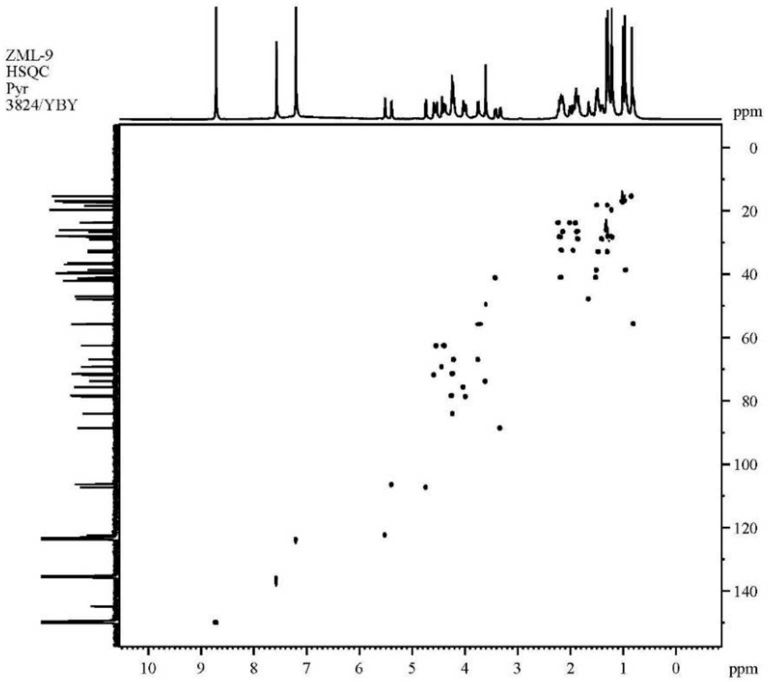

Figure S5. ^{1}H-^{1}H COSY spectrum of Acasentrioid A (**1**).

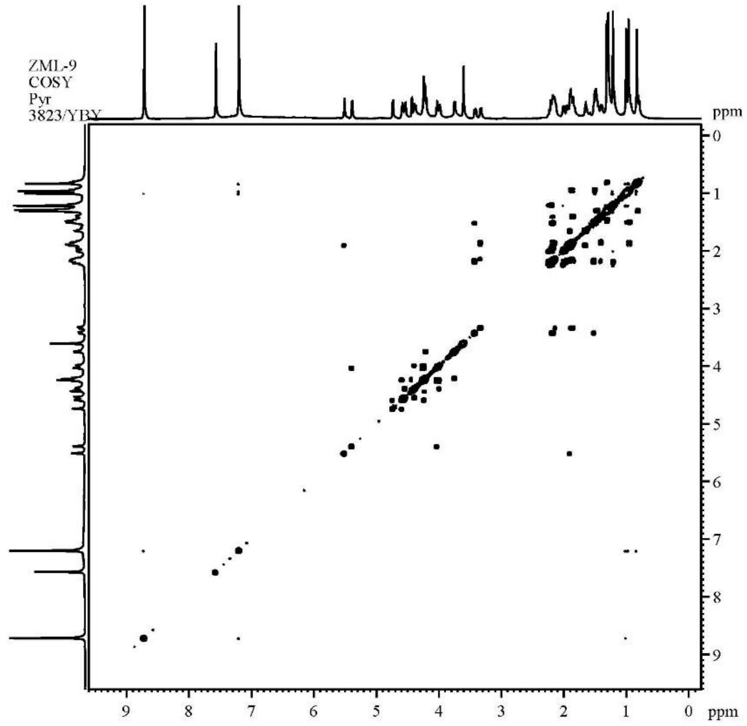

Figure S6. HMBC spectrum of Acasentrioid A (**1**).

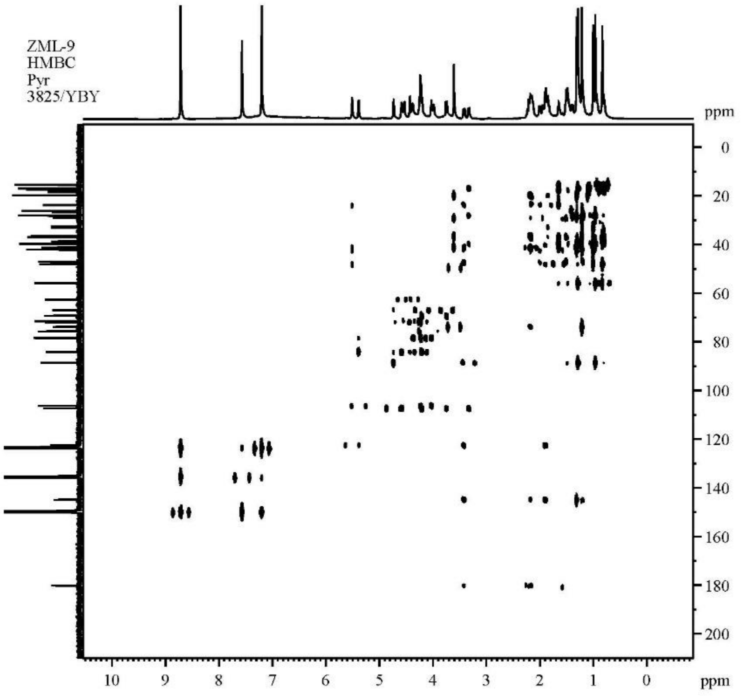

Figure S7. NOESY spectrum of Acasentrioid A (**1**).

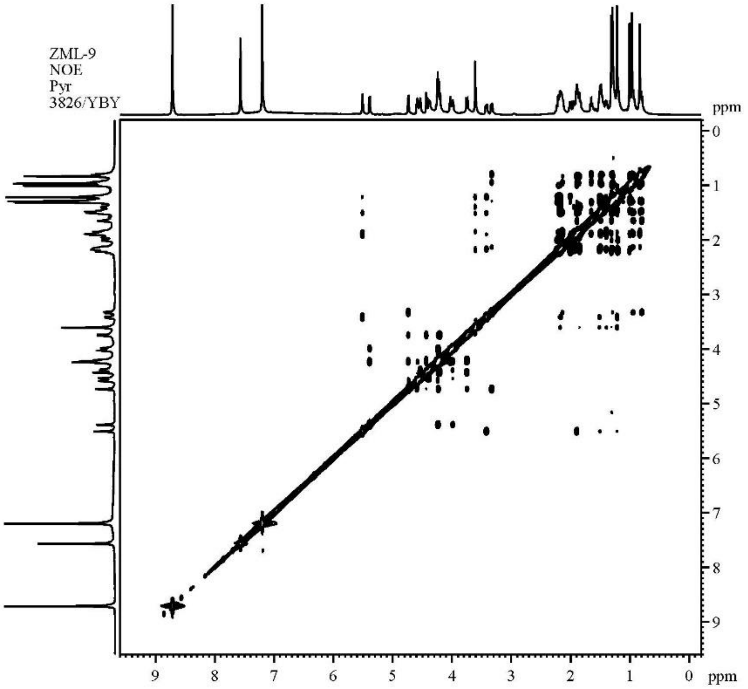

Figure S8. HR-ESI-MS spectrum of Acasentrioid A (**1**).

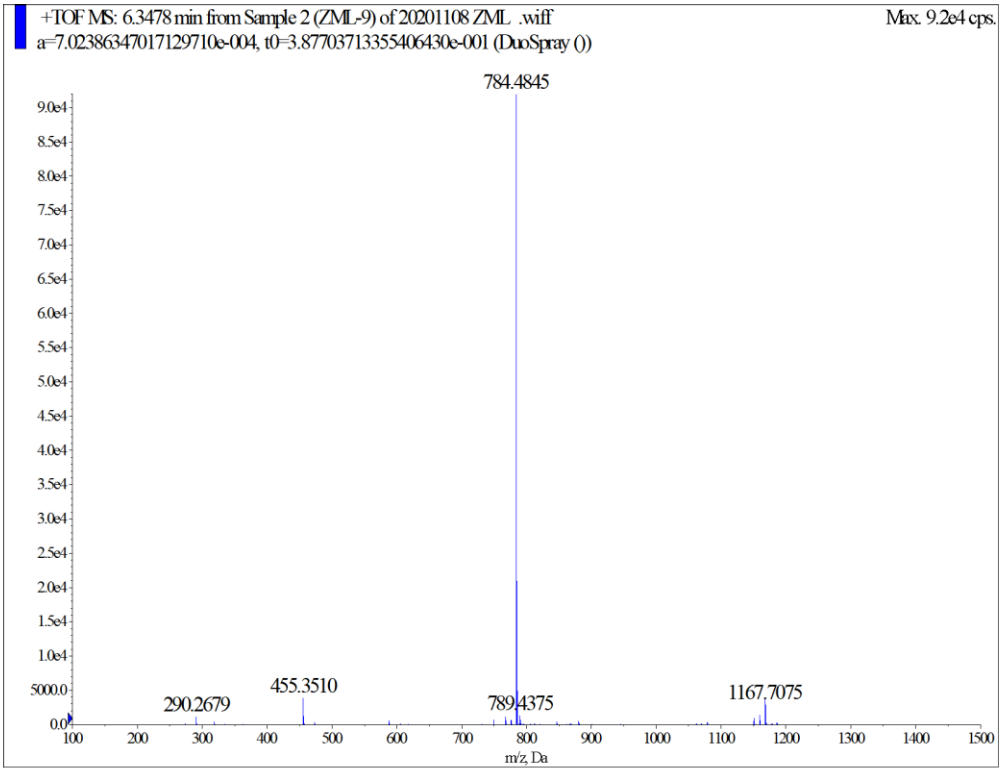

Figure S9. CD spectrum of Acasentrioid A (**1**).

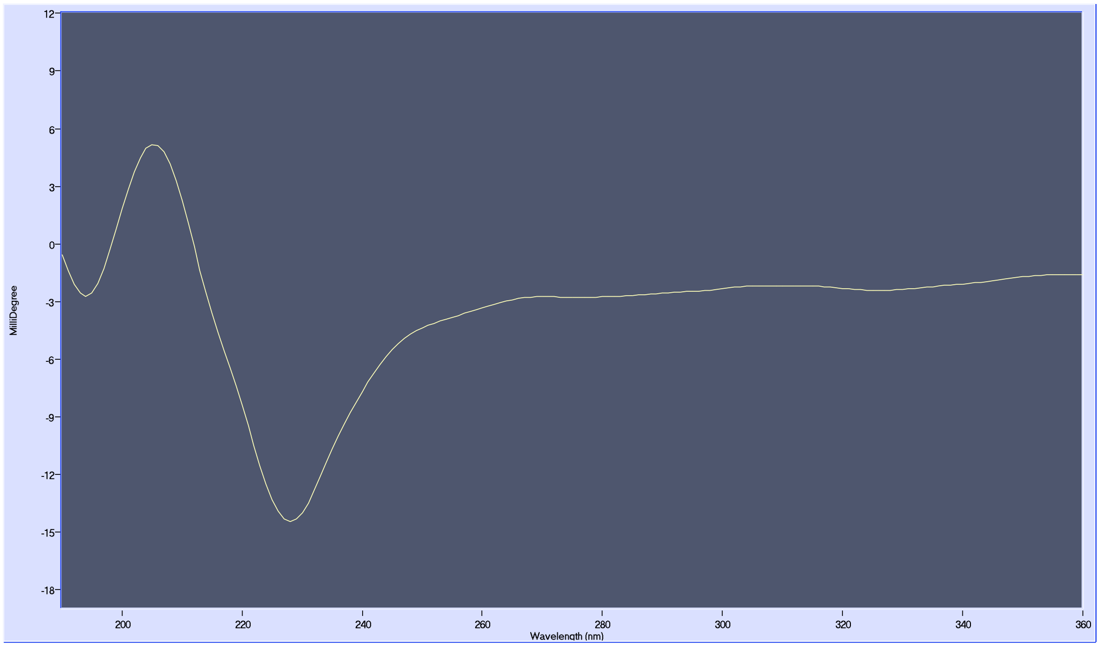

Figure S10. IR spectrum of Acasentrioid A (**1**).

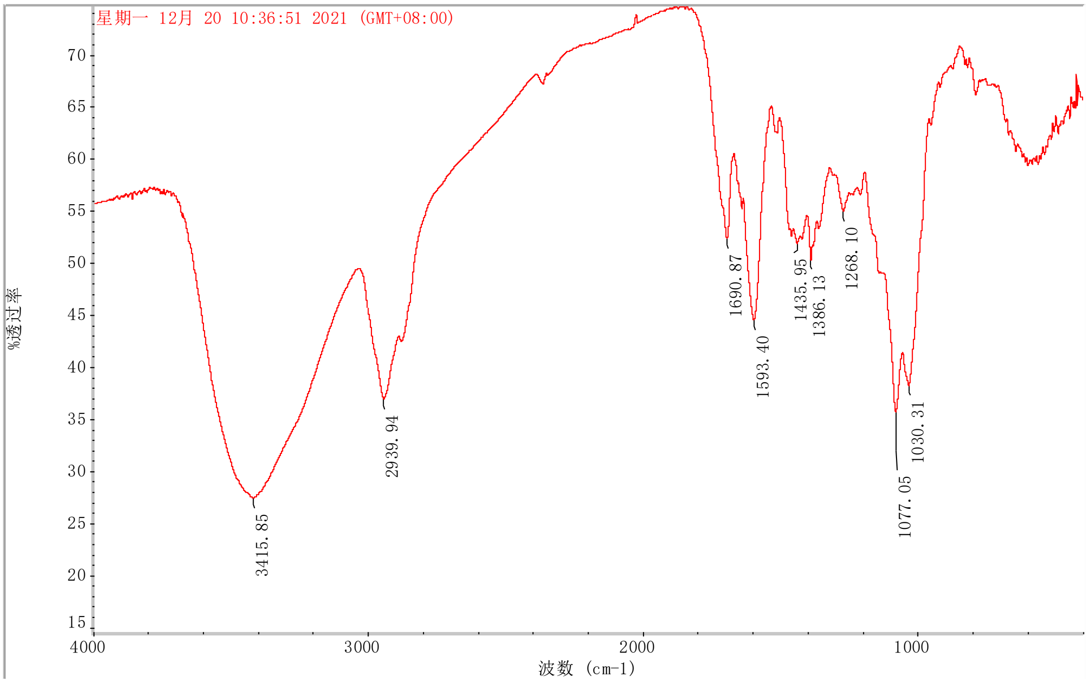

Figure S11. ^{1}H NMR spectrum of Acasentrioid B (**2**) (600 MHz, pyridine-d_5).

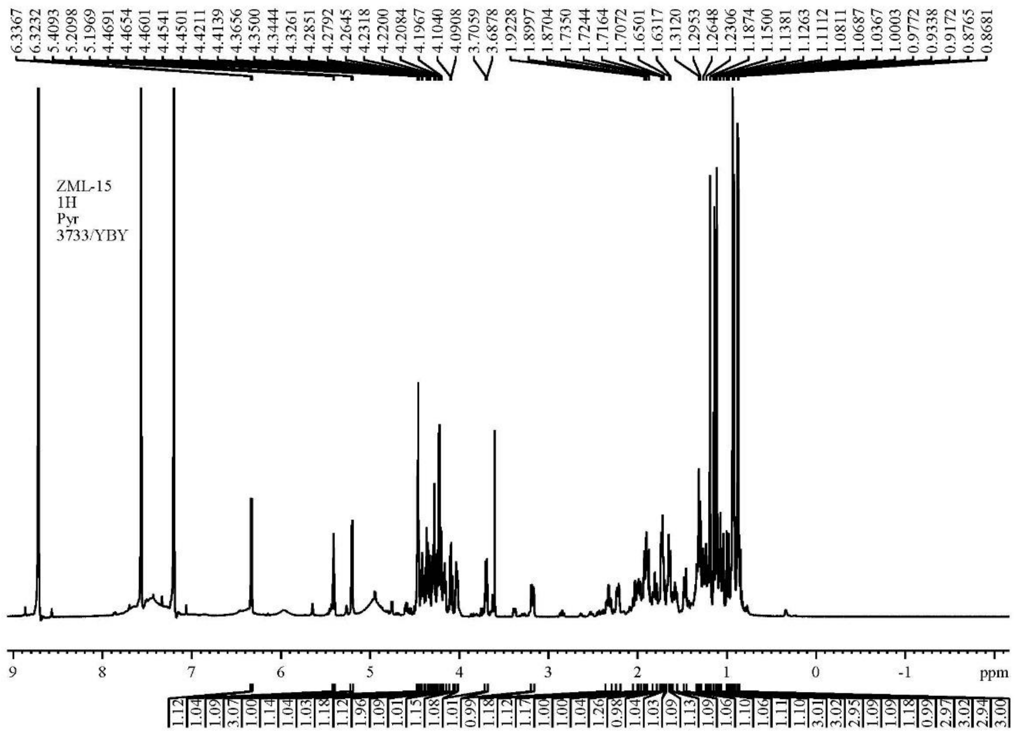

Figure S12. ^{13}C NMR spectrum of Acasentrioid B (**2**) (150 MHz, pyridine-d_5).

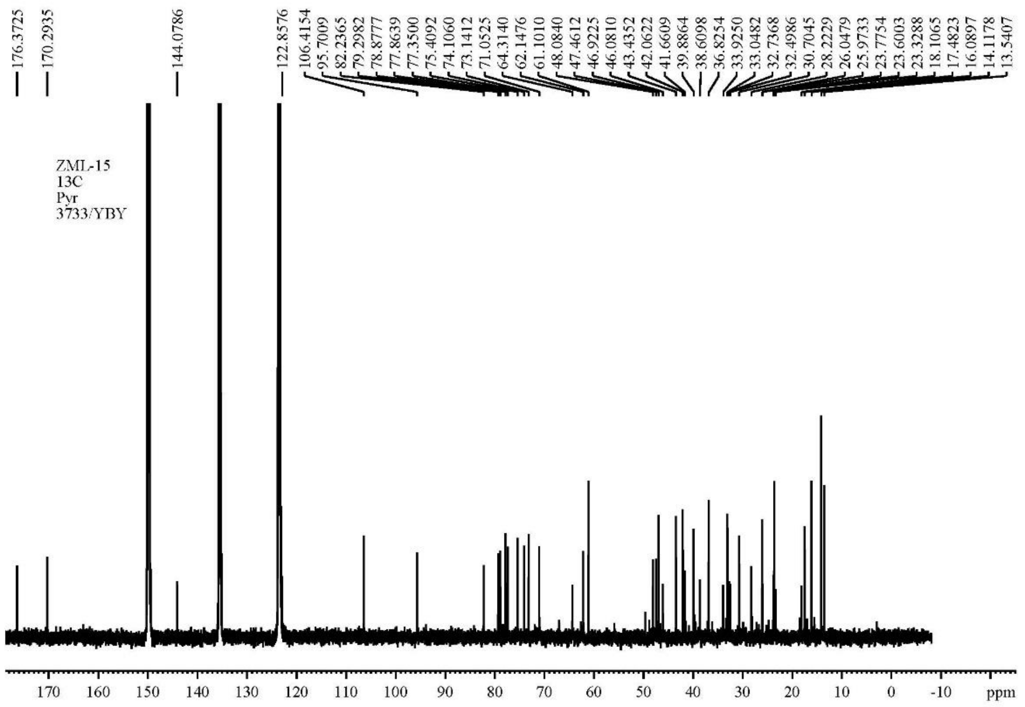

Figure S13. DEPT spectrum of Acasentrioid B (**2**).

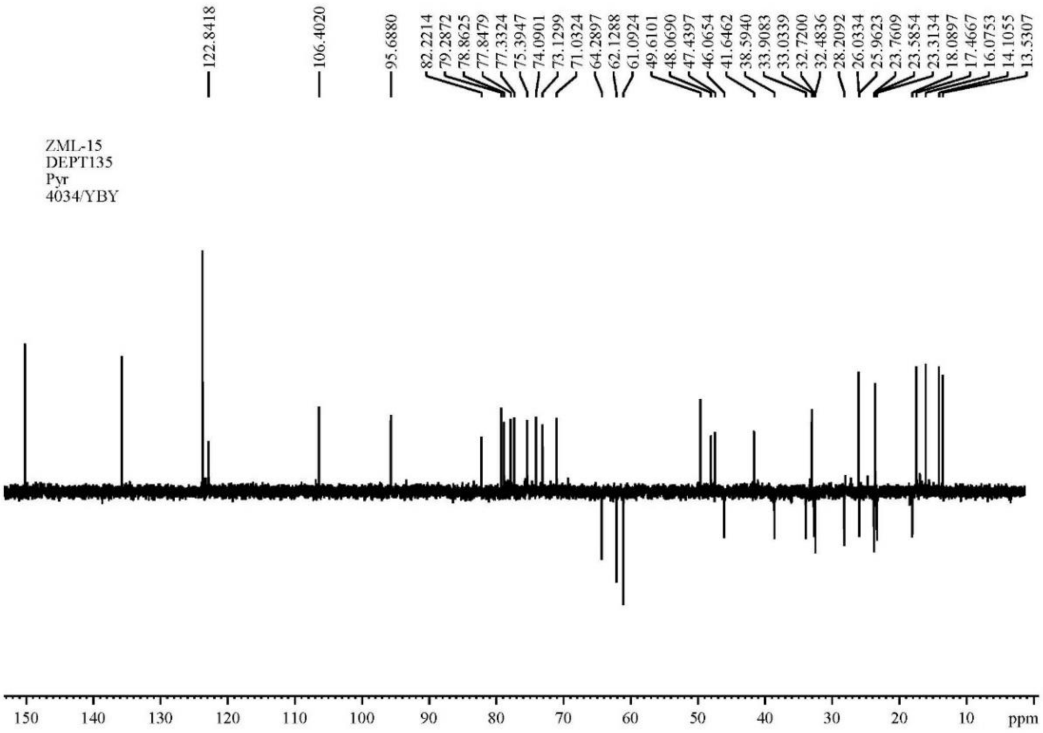

Figure S14. HSQC spectrum of Acasentrioid B (**2**).

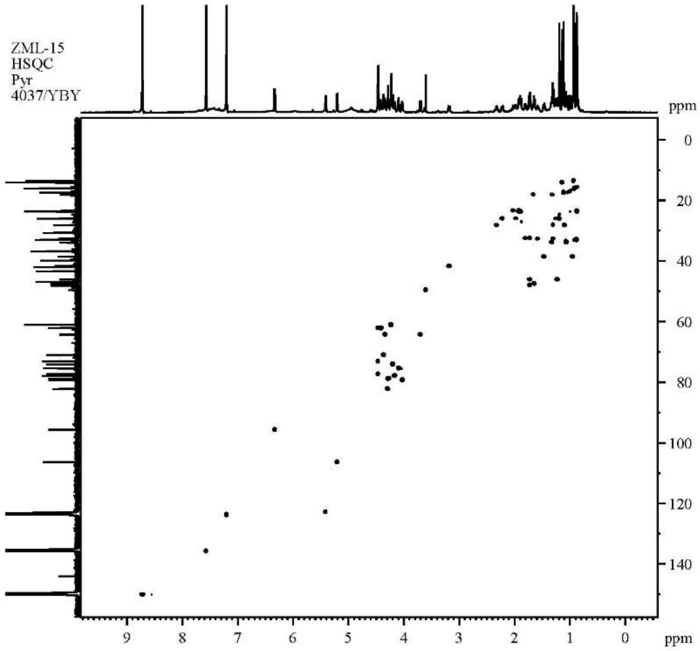

Figure S15. ^{1}H-^{1}H COSY spectrum of Acasentrioid B (**2**).

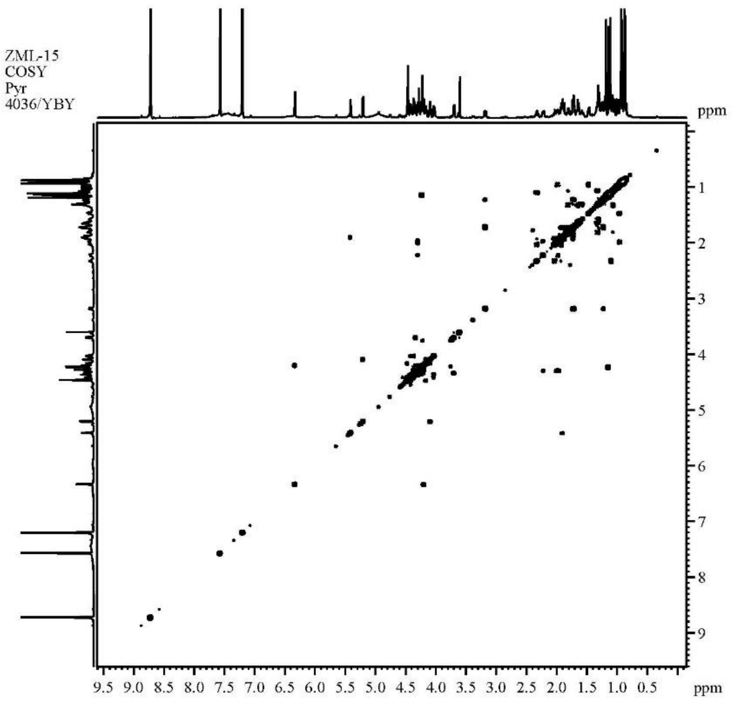

Figure S16. HMBC spectrum of Acasentrioid B (**2**).

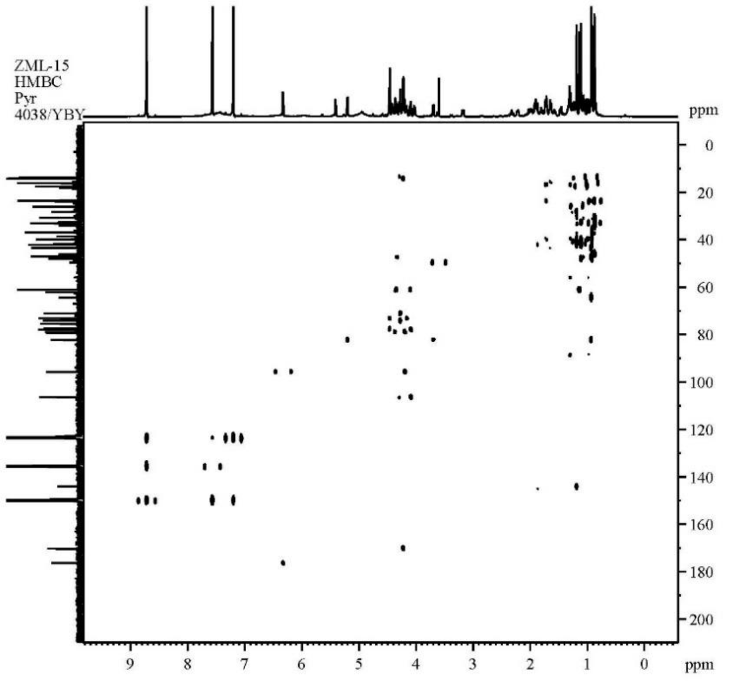

Figure S17. NOESY spectrum of Acasentrioid B (**2**).

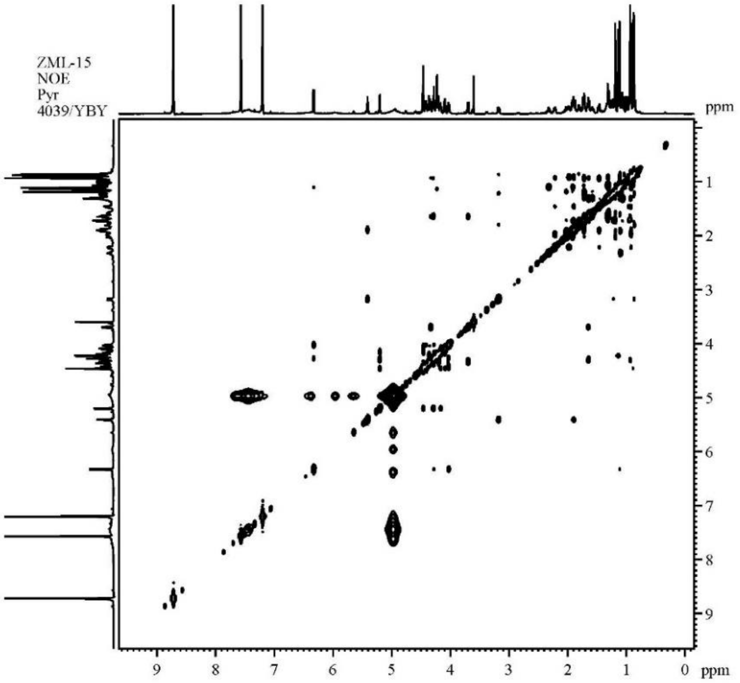

Figure S18. HR-ESI-MS spectrum of Acasentrioid B (**2**).

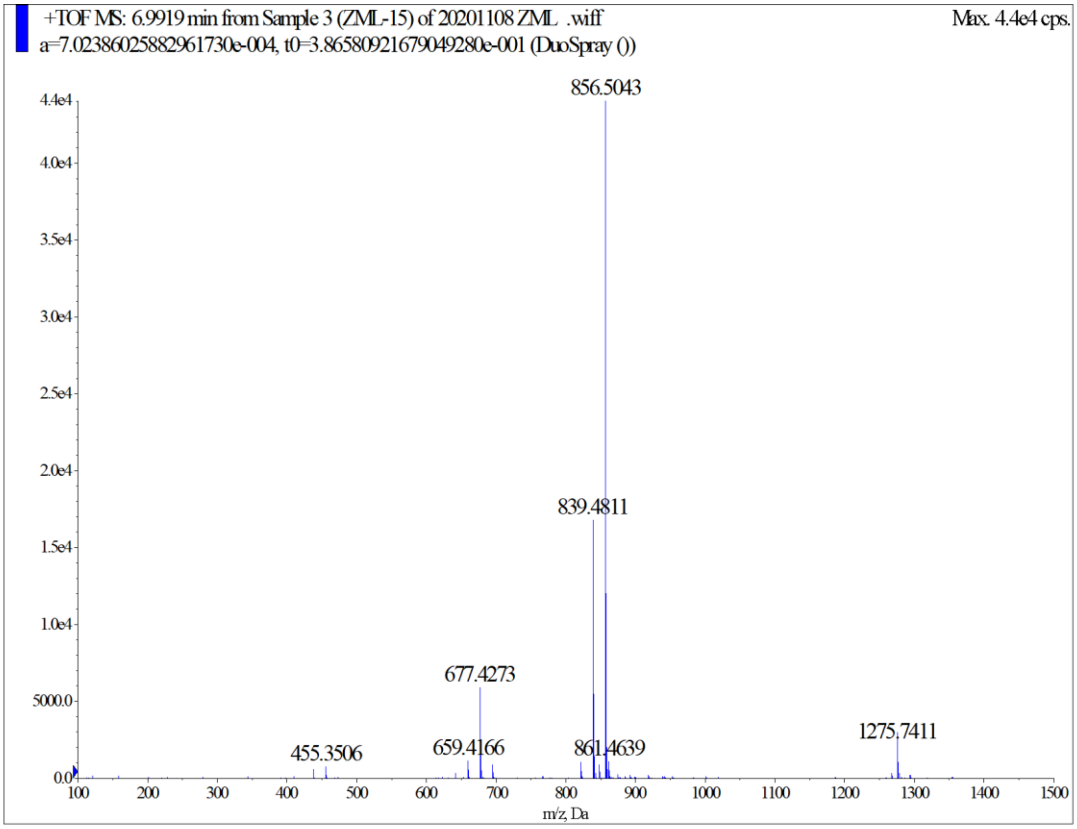

Figure S19. CD spectrum of Acasentrioid B (**2**).

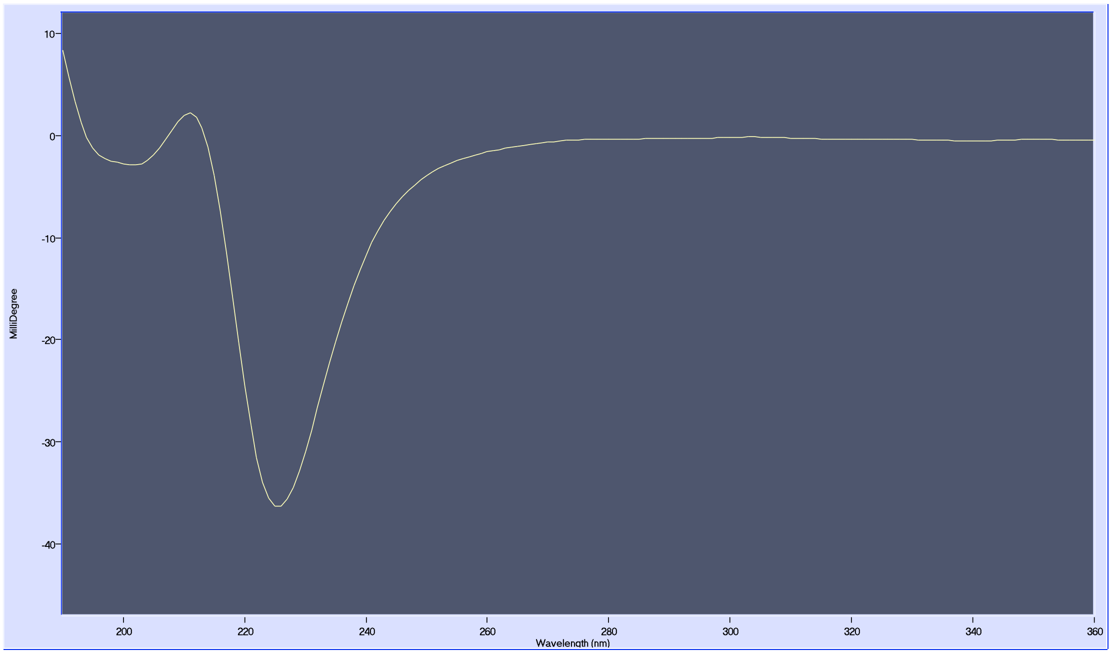

Figure S20. IR spectrum of Acasentrioid B (**2**).

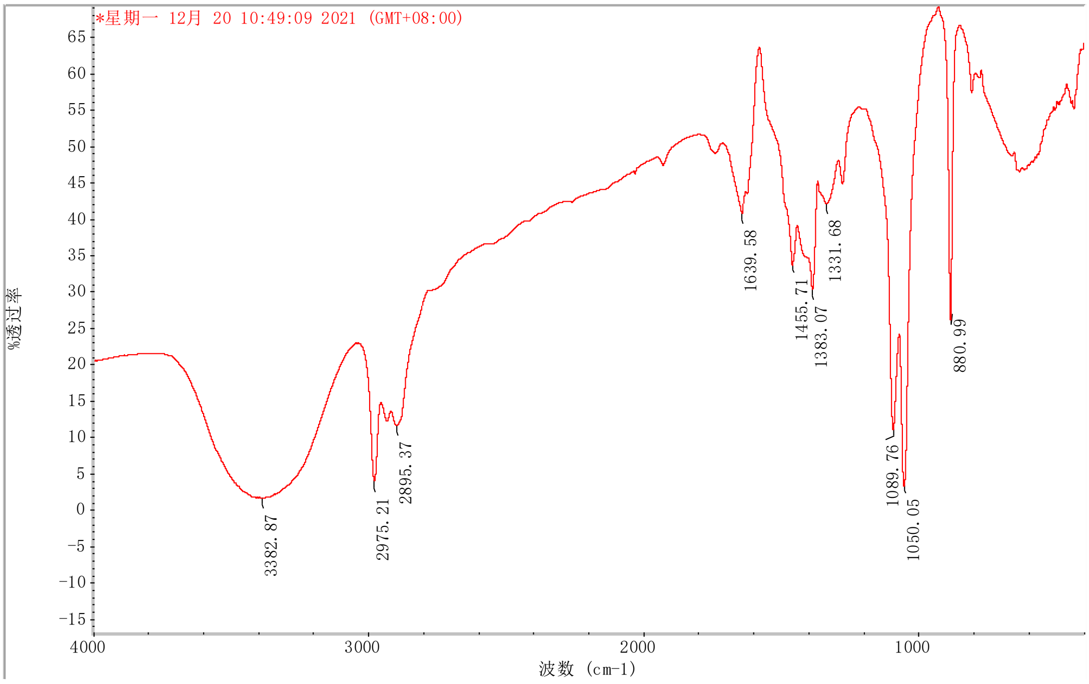

Figure S21. ^{1}H NMR spectrum of Acasentrioid C (**3**) (600 MHz, pyridine-d_5).

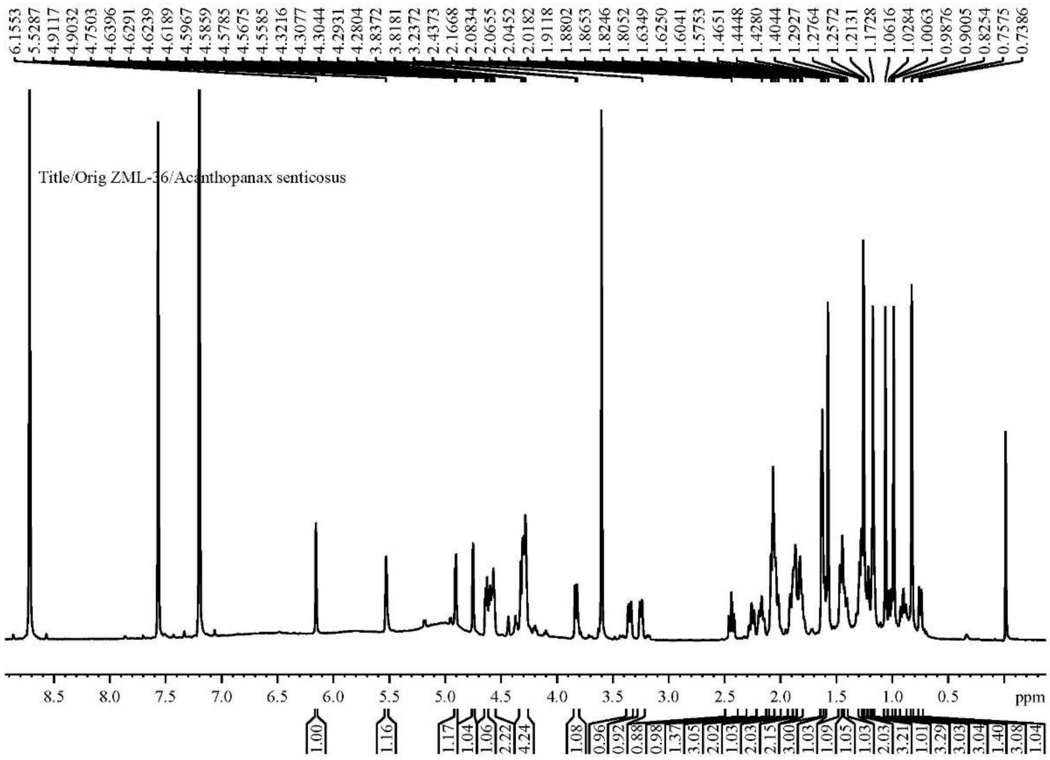

Figure S22. ^{13}C NMR spectrum of Acasentrioid C (**3**) (150 MHz, pyridine-d_5).

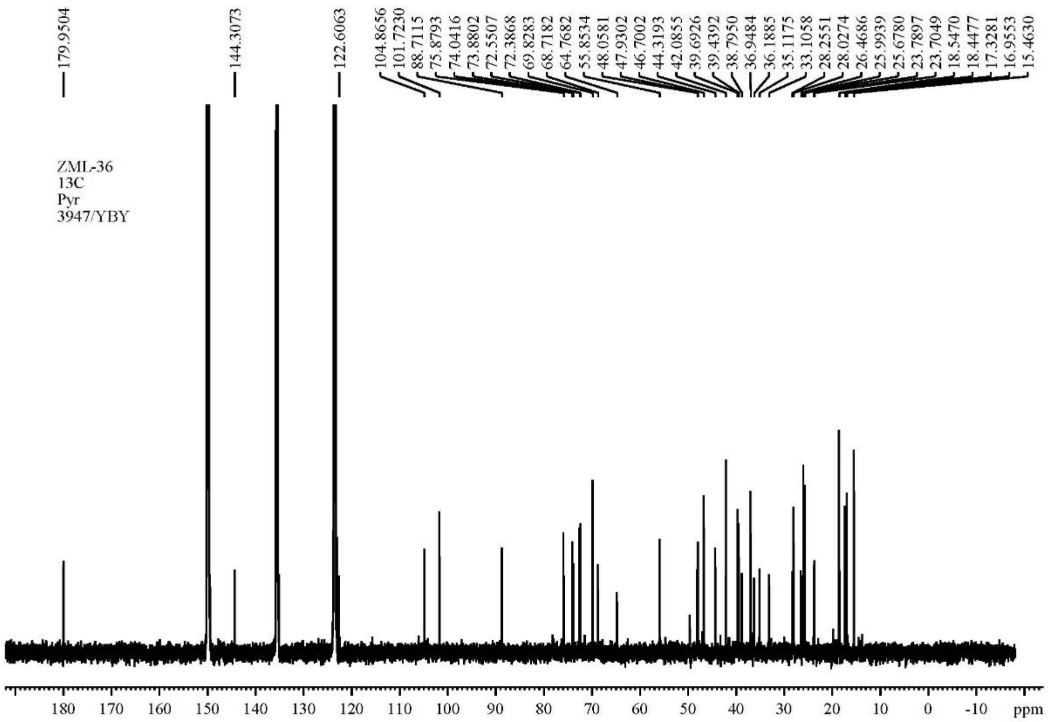

Figure S23. DEPT spectrum of Acasentrioid C (**3**).

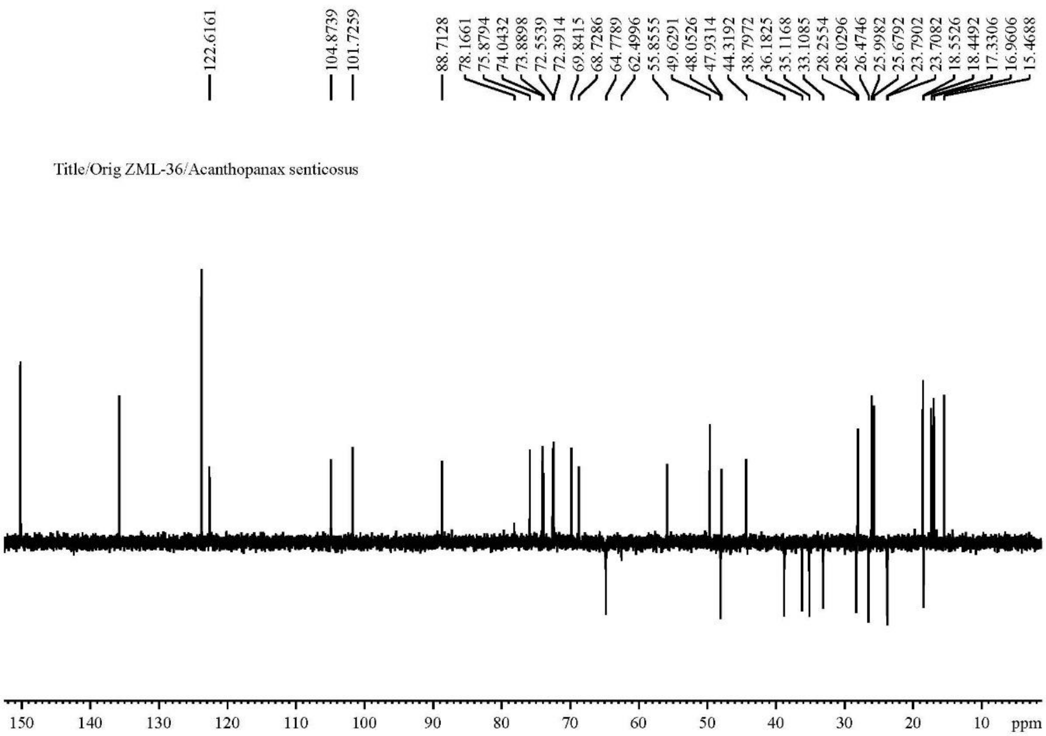

Figure S24. HSQC spectrum of Acasentrioid C (**3**).

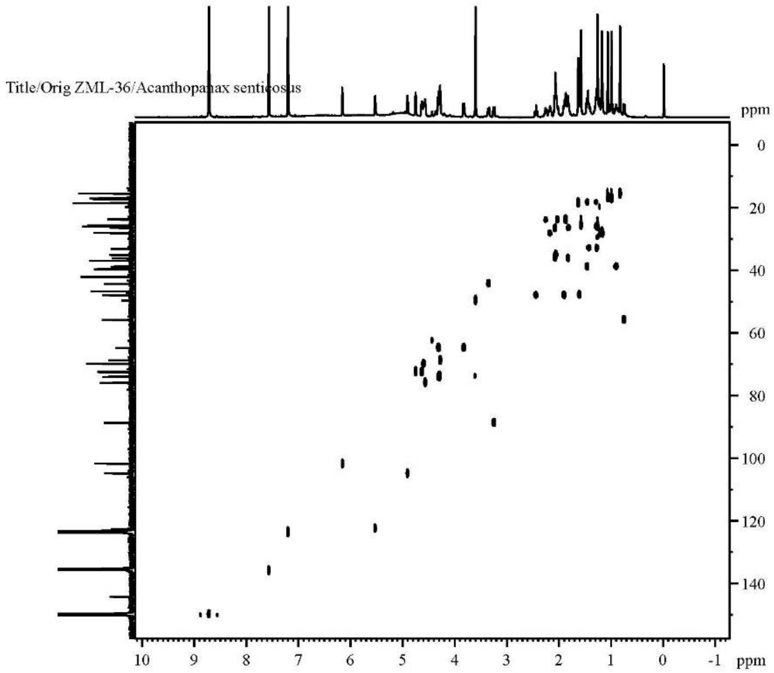

Figure S25. ^{1}H-^{1}H COSY spectrum of Acasentrioid C (**3**).

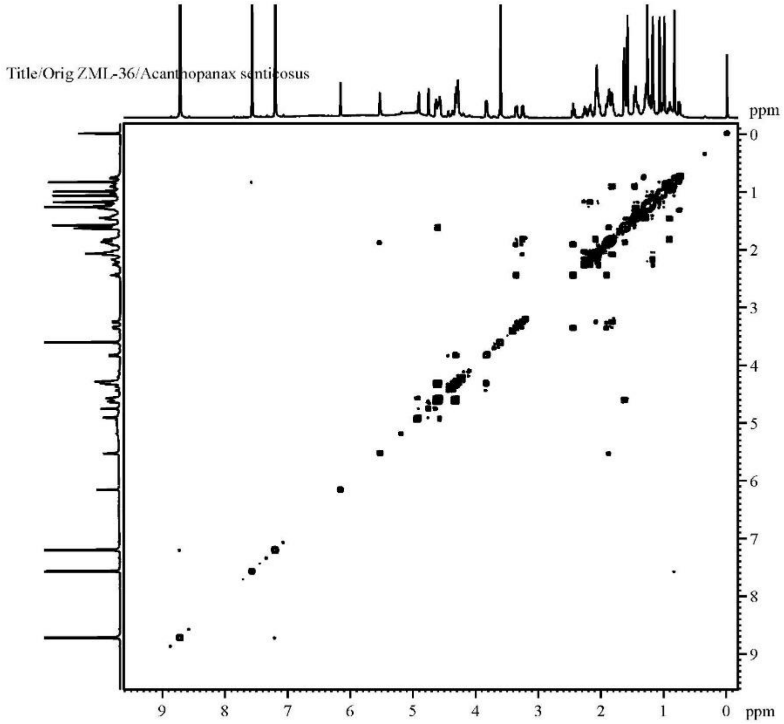

Figure S26. HMBC spectrum of Acasentrioid C (**3**).

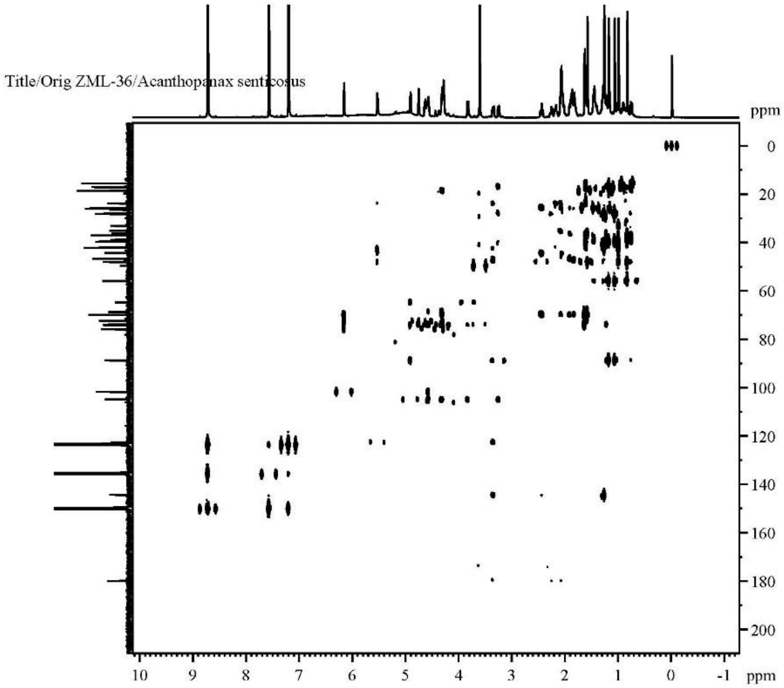

Figure S27. NOESY spectrum of Acasentrioid C (**3**).

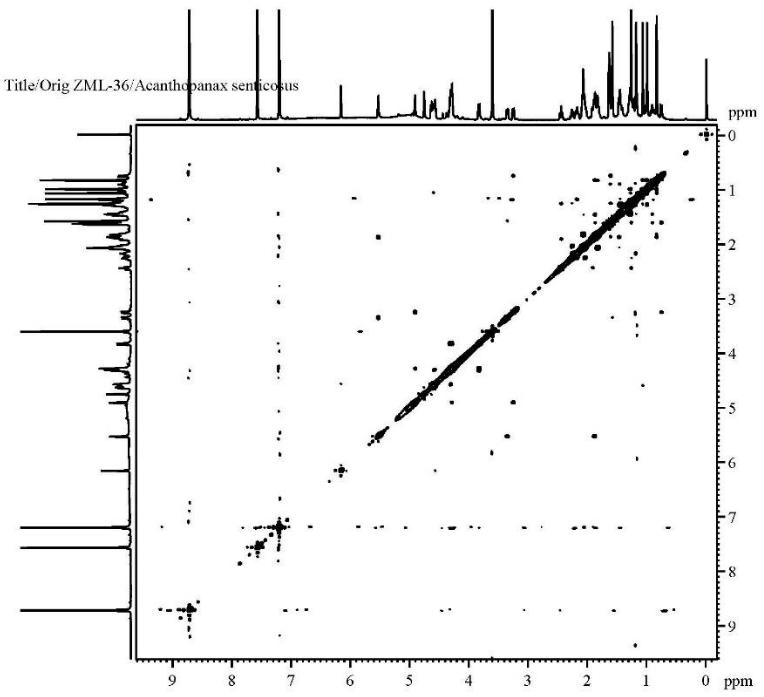

Figure S28. HR-ESI-MS spectrum of Acasentrioid C (**3**).

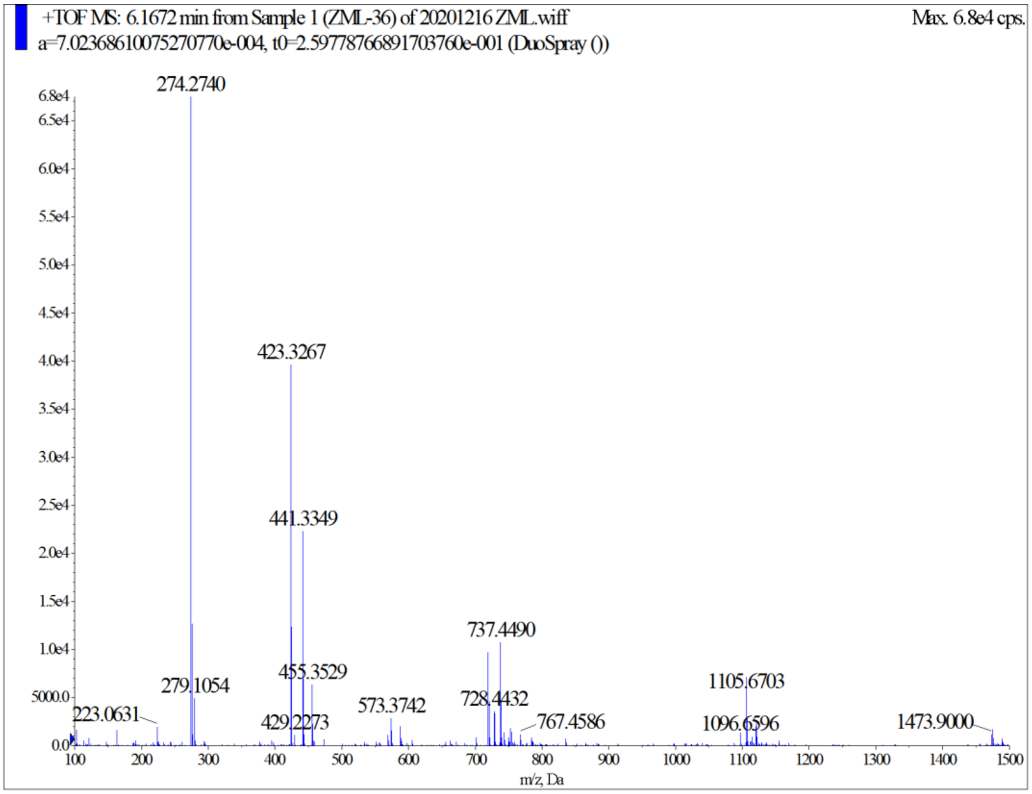

Figure S29. CD spectrum of Acasentrioid C (**3**).

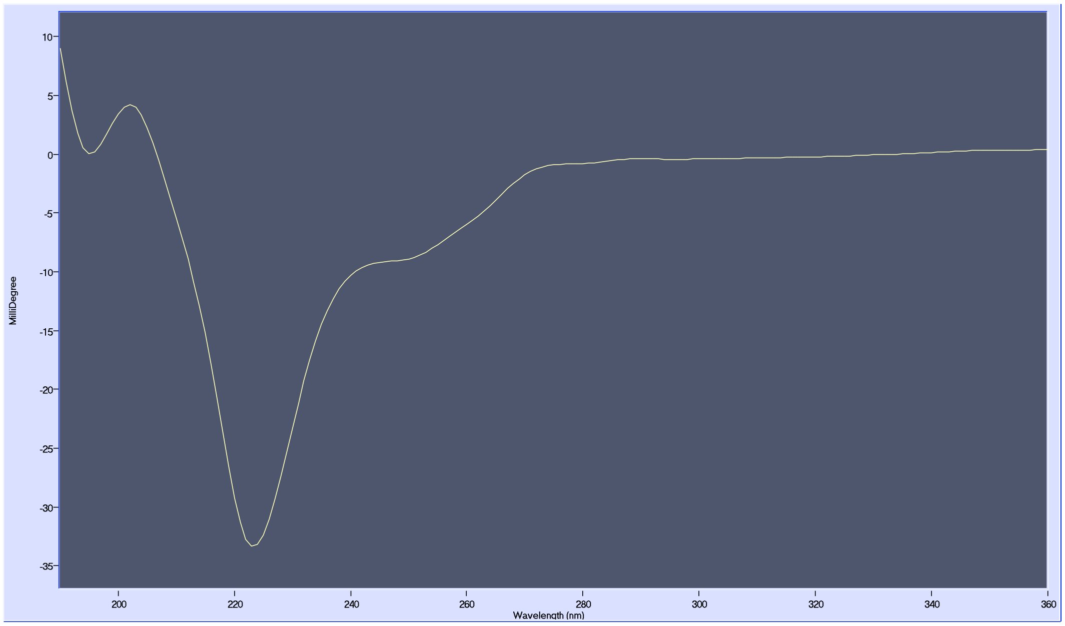

Figure S30. IR spectrum of Acasentrioid C (**3**).

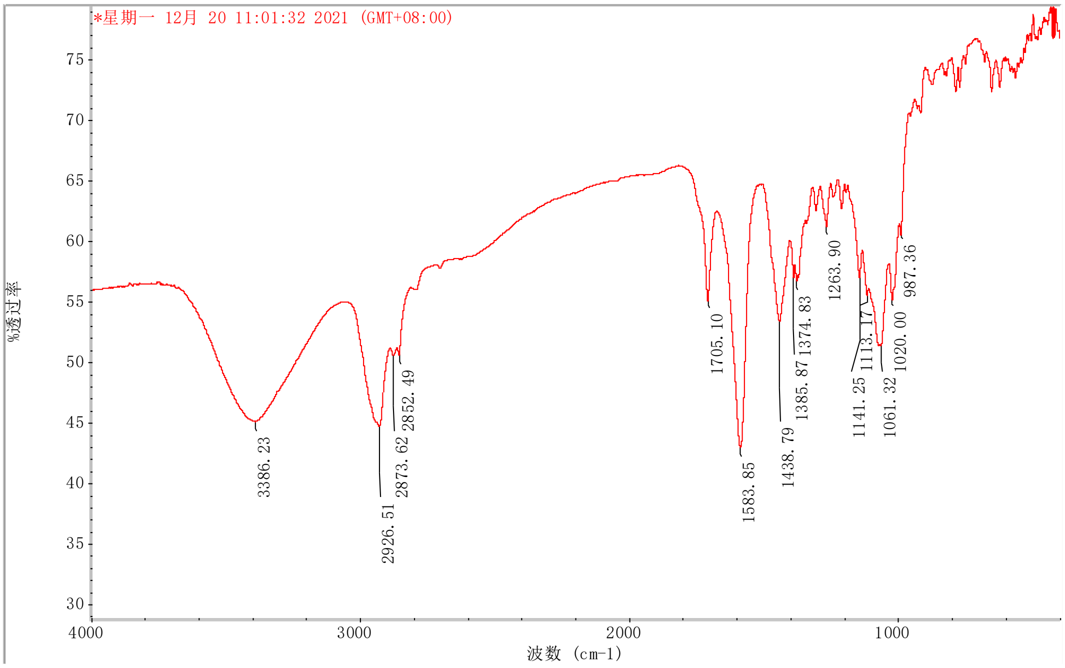

Figure S31. ^{1}H NMR spectrum of Acasentrioid D (**4**) (600 MHz, pyridine-d_5).

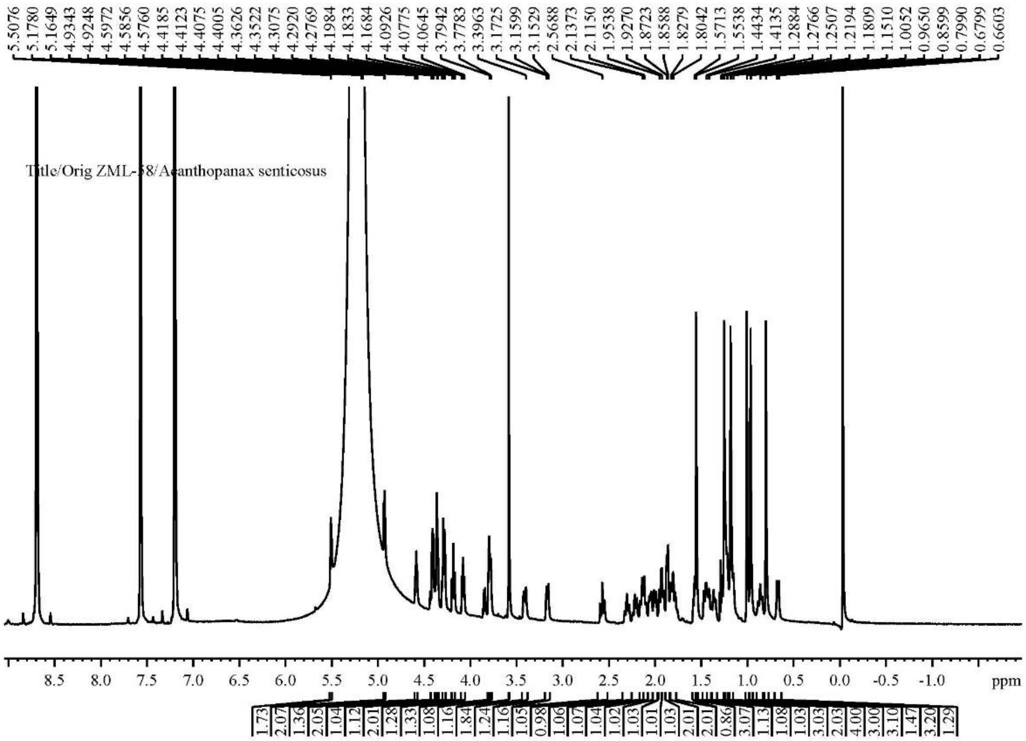

Figure S32. ^{13}C NMR spectrum of Acasentrioid D (**4**) (150 MHz, pyridine-d_5).

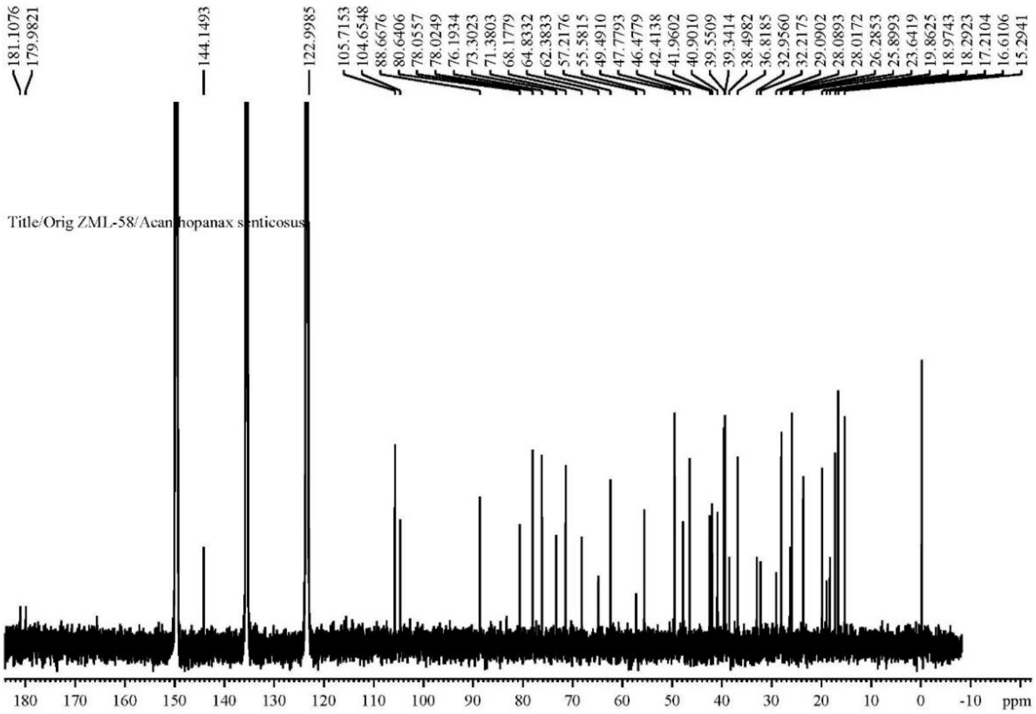

Figure S33. DEPT spectrum of Acasentrioid D (**4**).

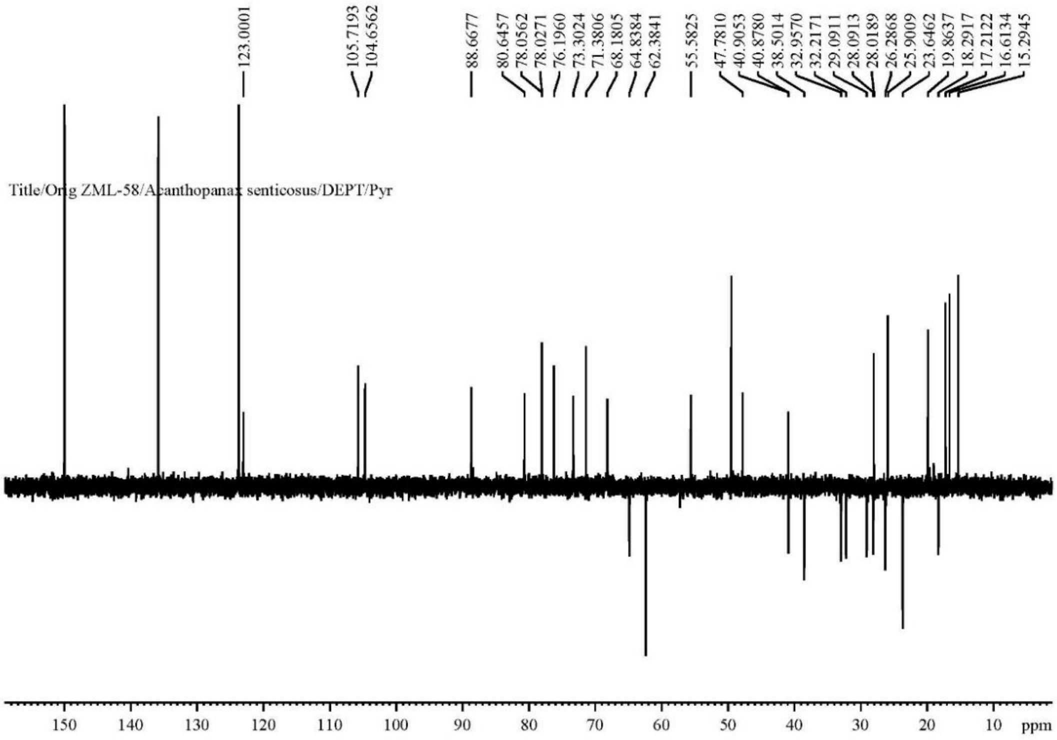

Figure S34. HSQC spectrum of Acasentrioid D (**4**).

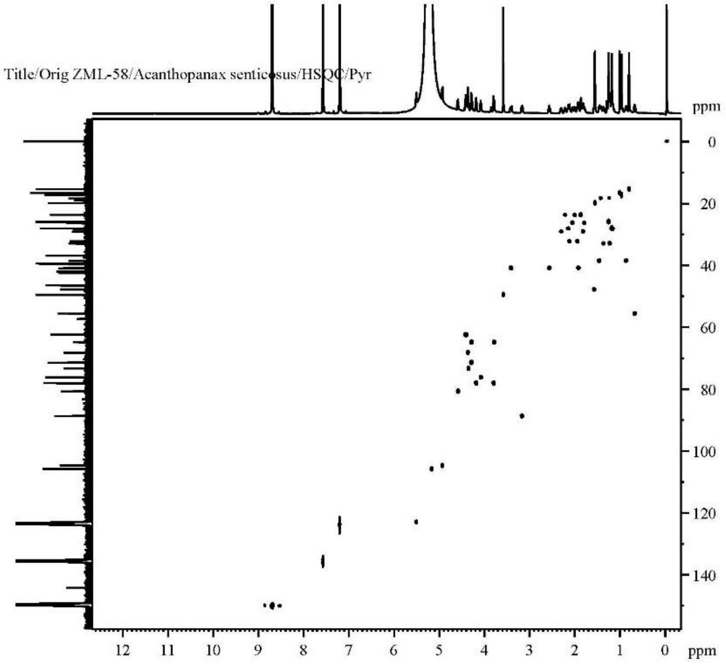

Figure S35. ^{1}H-^{1}H COSY spectrum of Acasentrioid D (**4**).

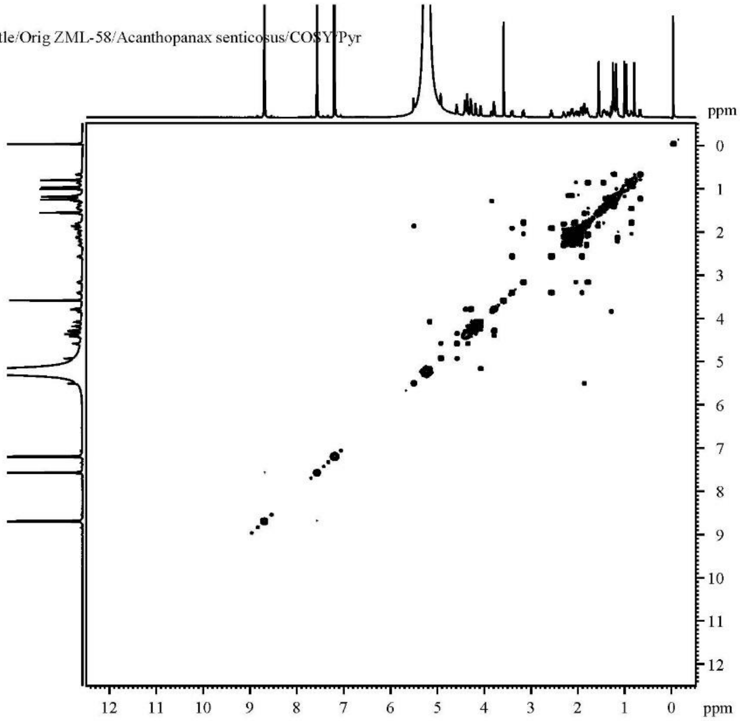

Figure S36. HMBC spectrum of Acasentrioid D (**4**).

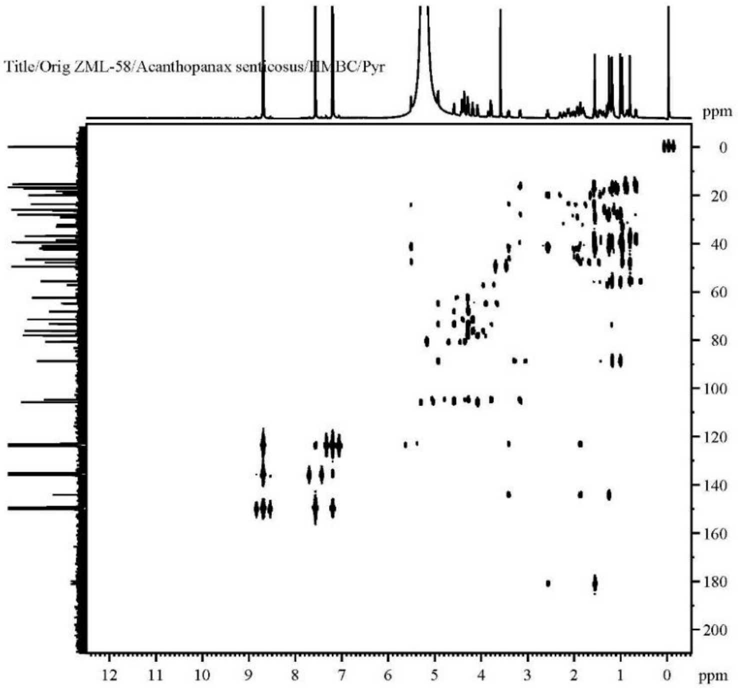

Figure S37. NOESY spectrum of Acasentrioid D (**4**).

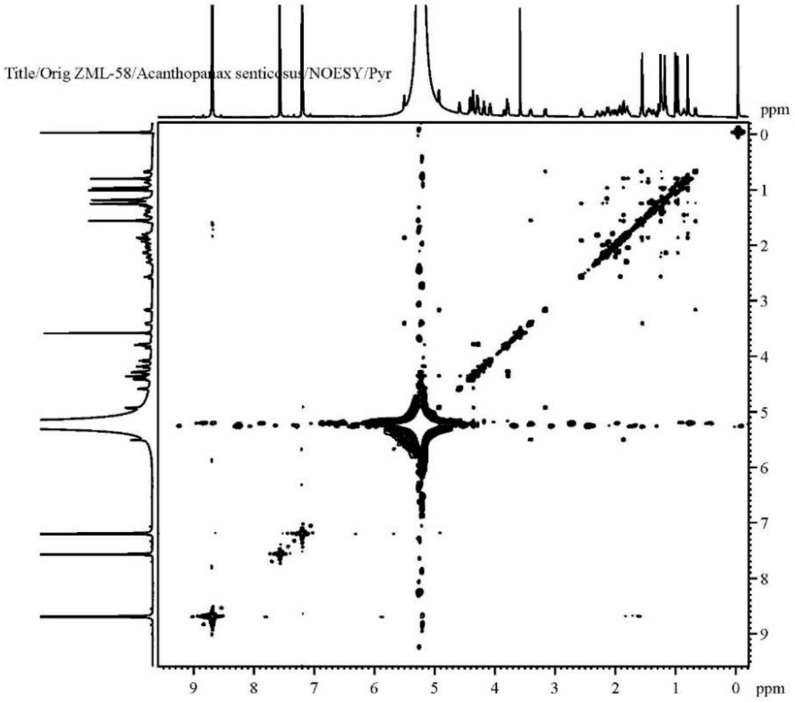

Figure S38. HR-ESI-MS spectrum of Acasentrioid D (**4**).

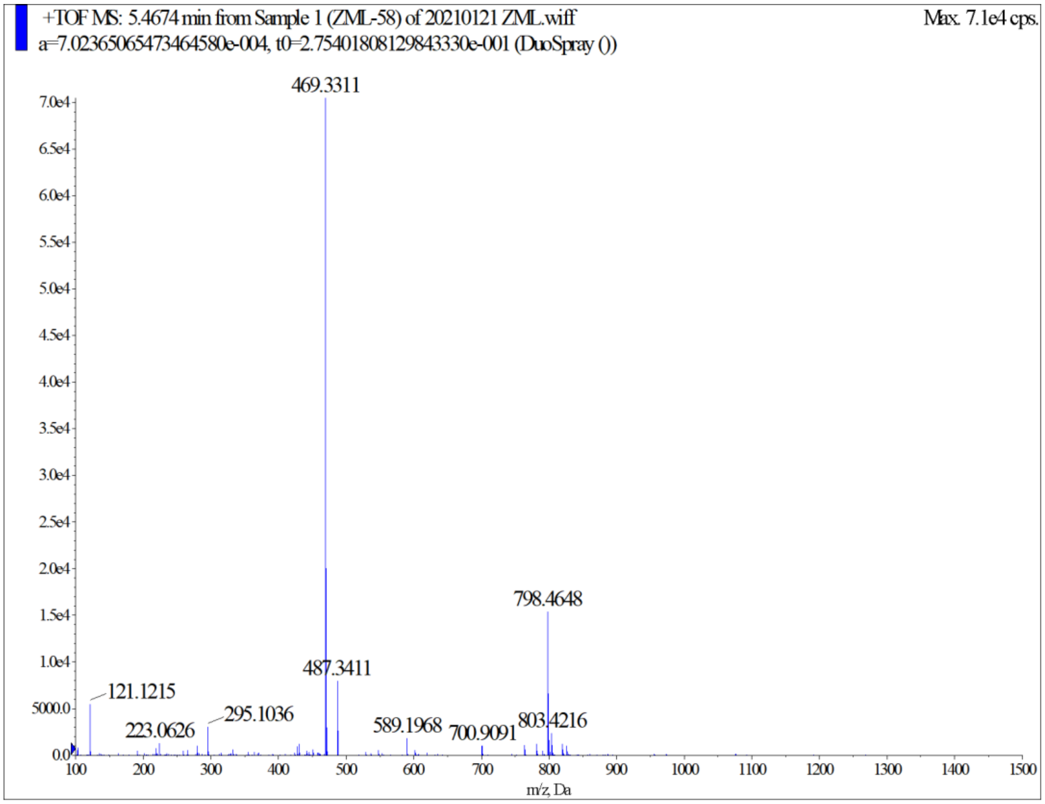

Figure S39. CD spectrum of Acasentrioid D (**4**).

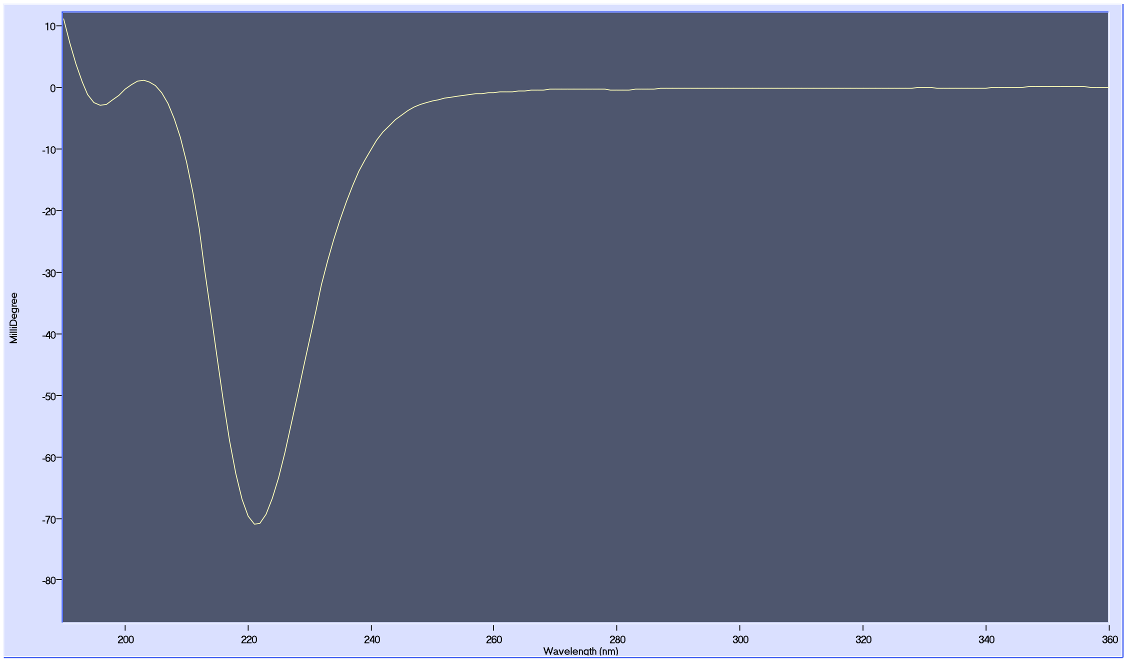

Figure S40. IR spectrum of Acasentrioid D (**4**).

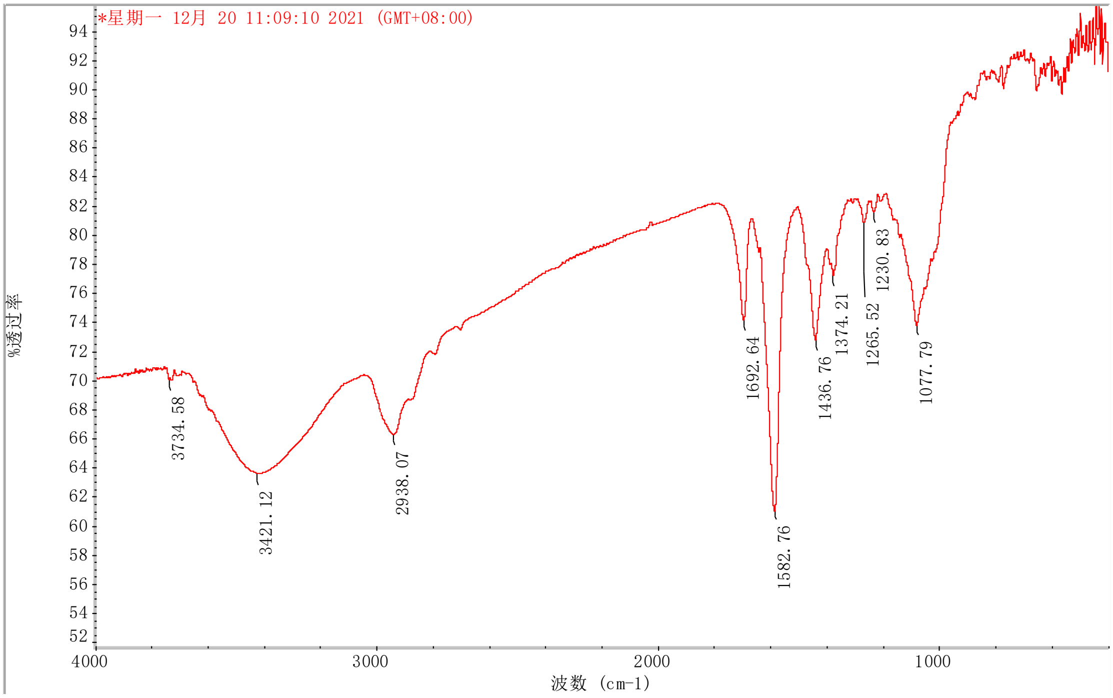

Figure S41. ^{1}H NMR spectrum of Acasentrioid E (**5**) (600 MHz, pyridine-d_5).

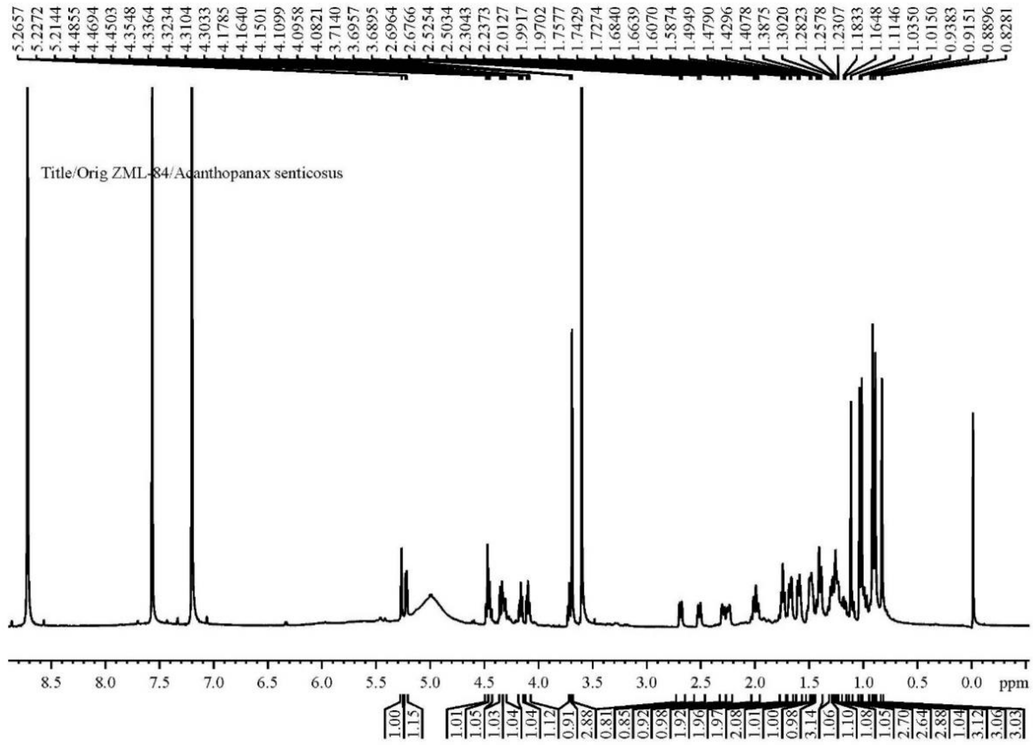

Figure S42. ^{13}C NMR spectrum of Acasentrioid E (**5**) (150 MHz, pyridine-d_5).

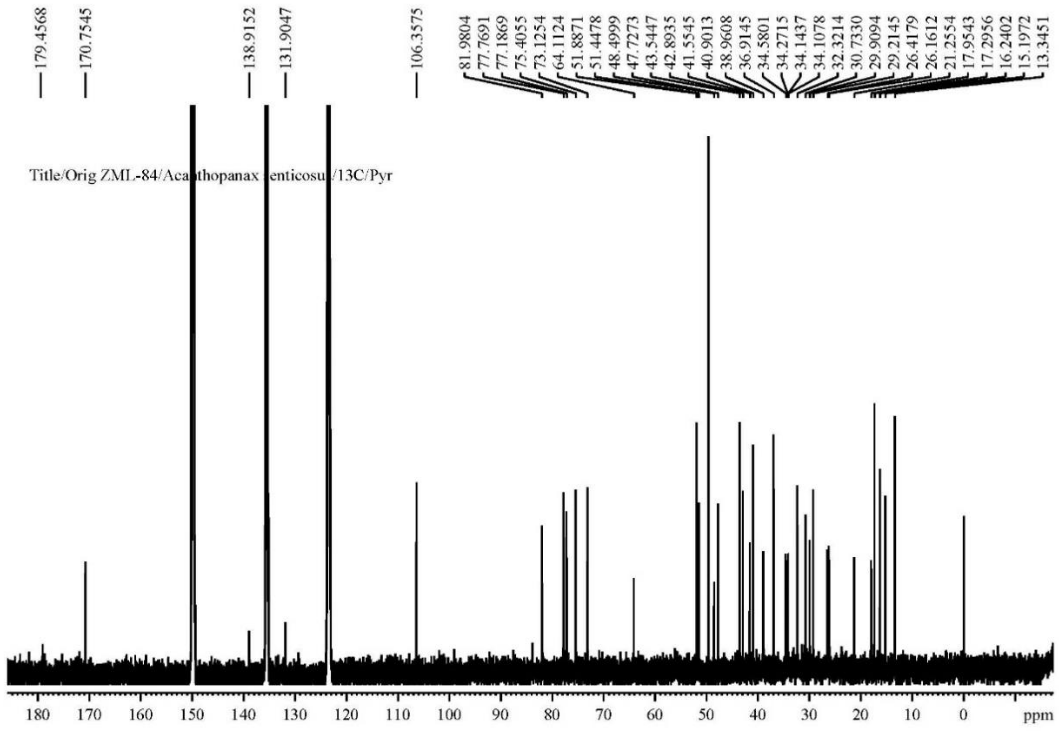

Figure S43. DEPT spectrum of Acasentrioid E (**5**).

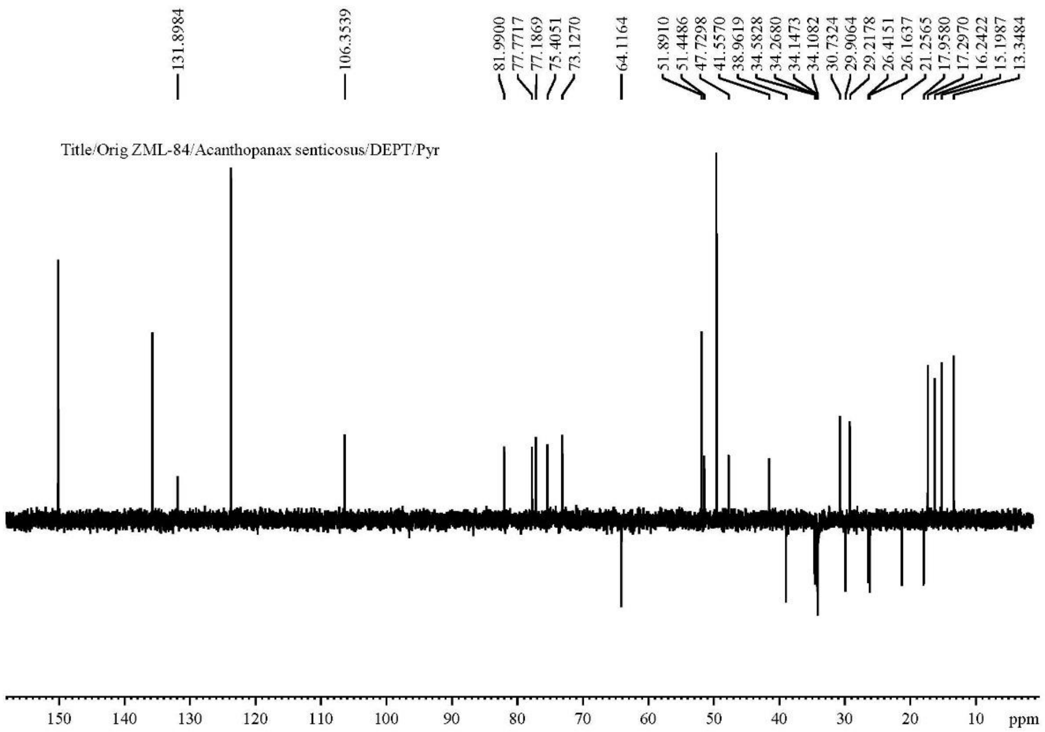

Figure S44. HSQC spectrum of Acasentrioid E (**5**).

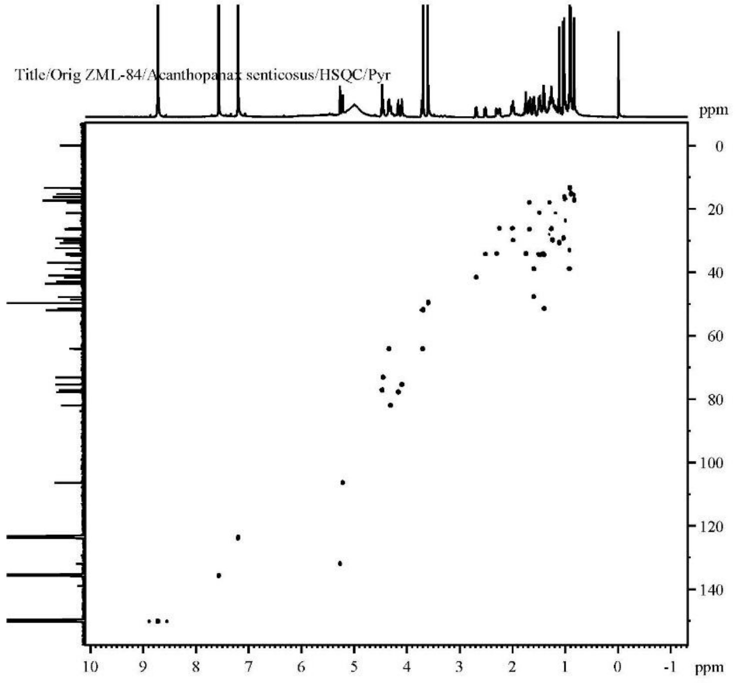

Figure S45. ^{1}H-^{1}H COSY spectrum of Acasentrioid E (**5**).

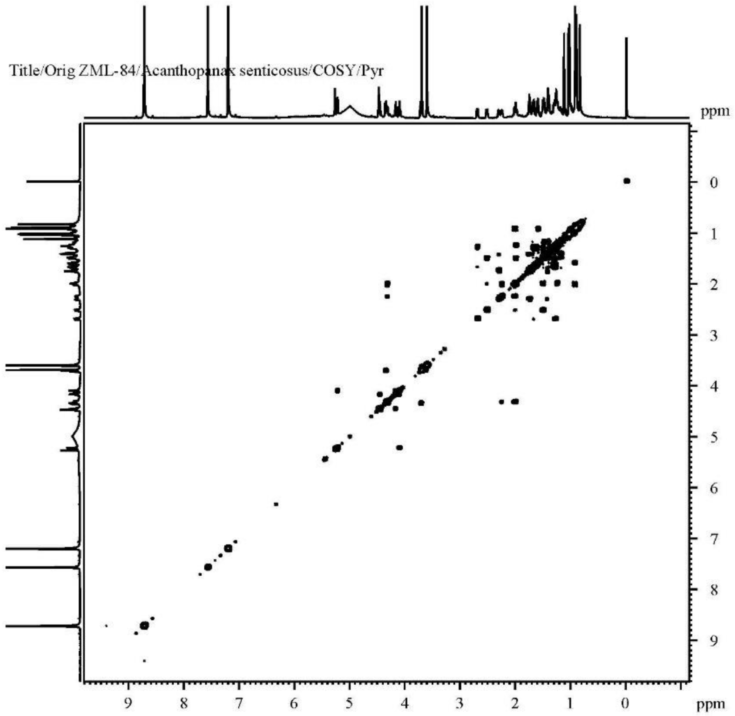

Figure S46. HMBC spectrum of Acasentrioid E (**5**).

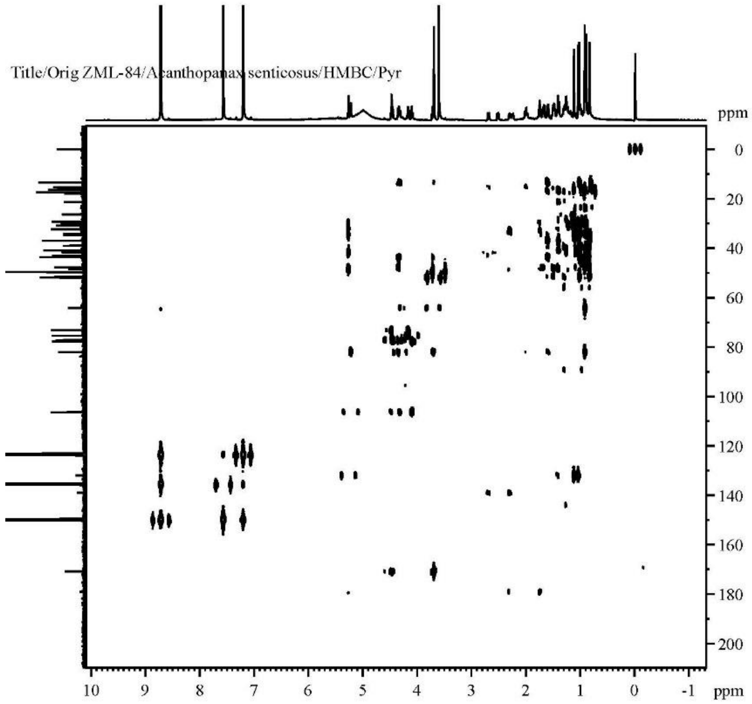

Figure S47. NOESY spectrum of Acasentrioid E (**5**).

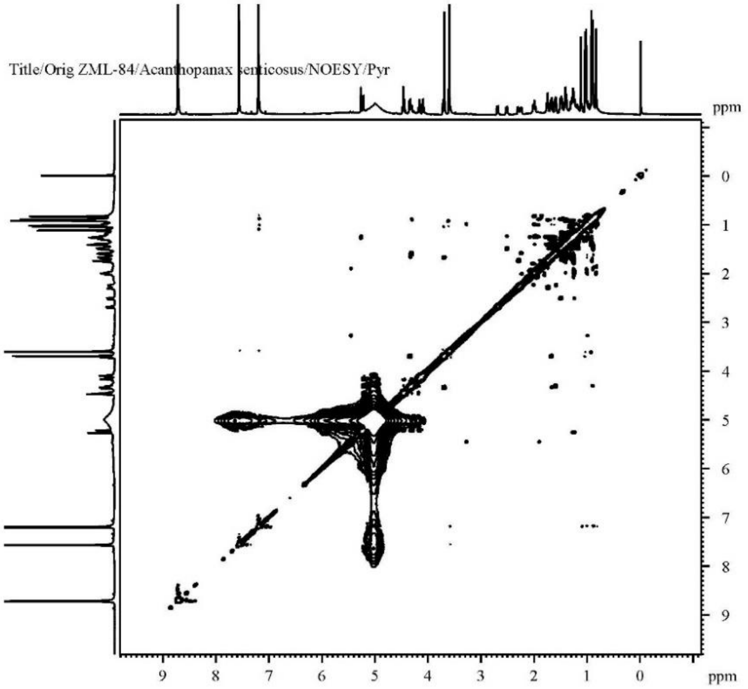

Figure S48. HR-ESI-MS spectrum of Acasentrioid E (**5**).

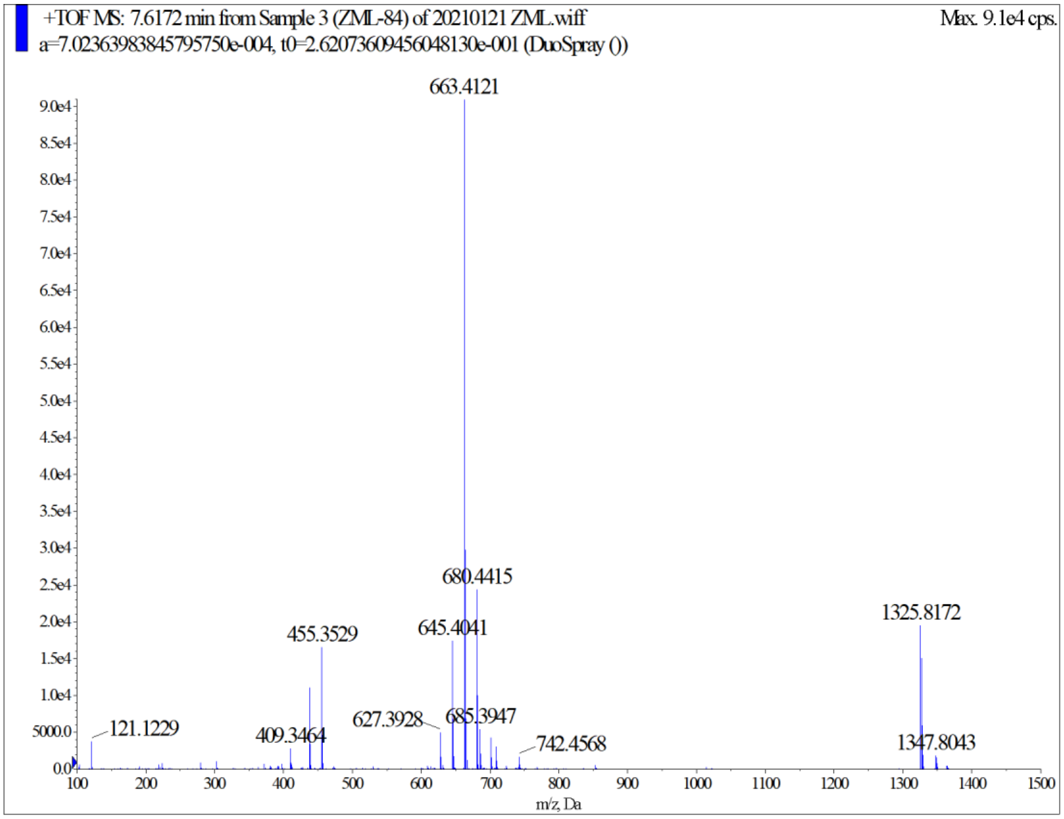

Figure S49. CD spectrum of Acasentrioid E (5).

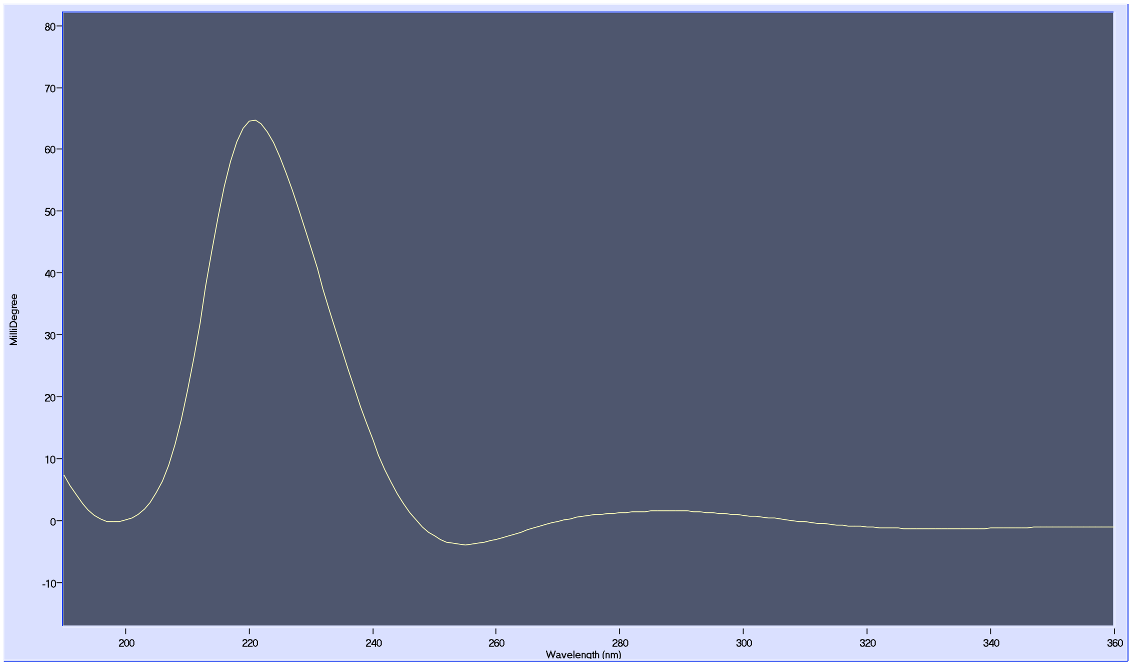

Figure S50. IR spectrum of Acasentrioid E (**5**).

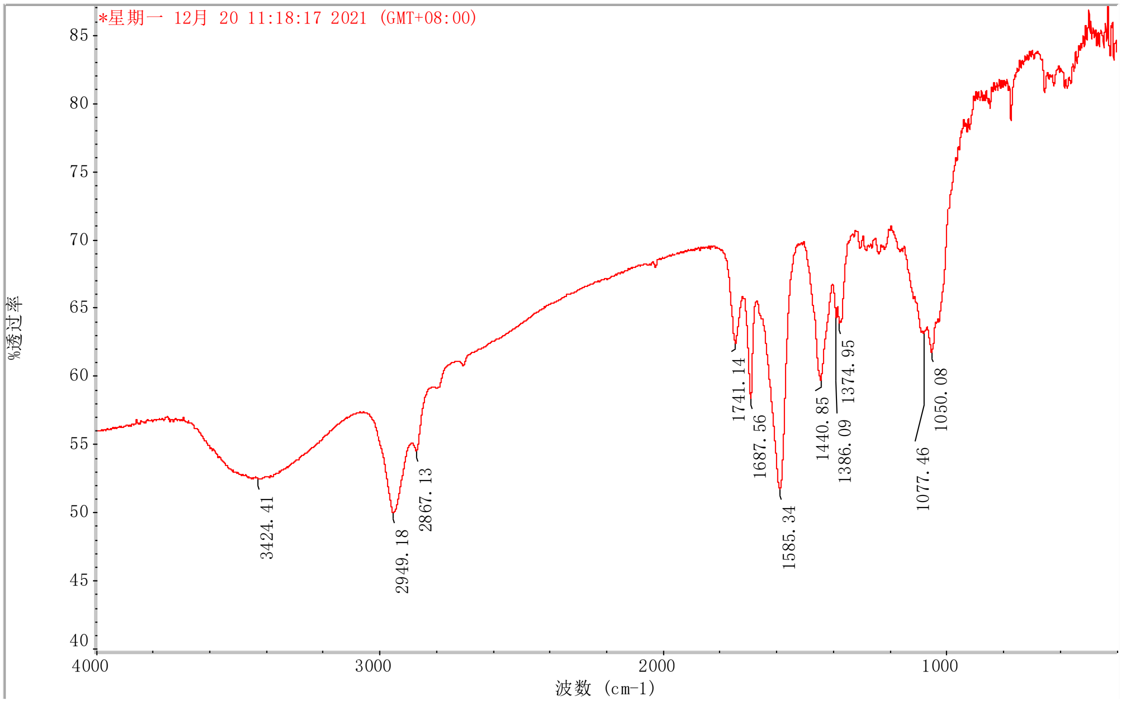

Figure S51. GC-MS analysis data of compound (**1**) hydrolysis

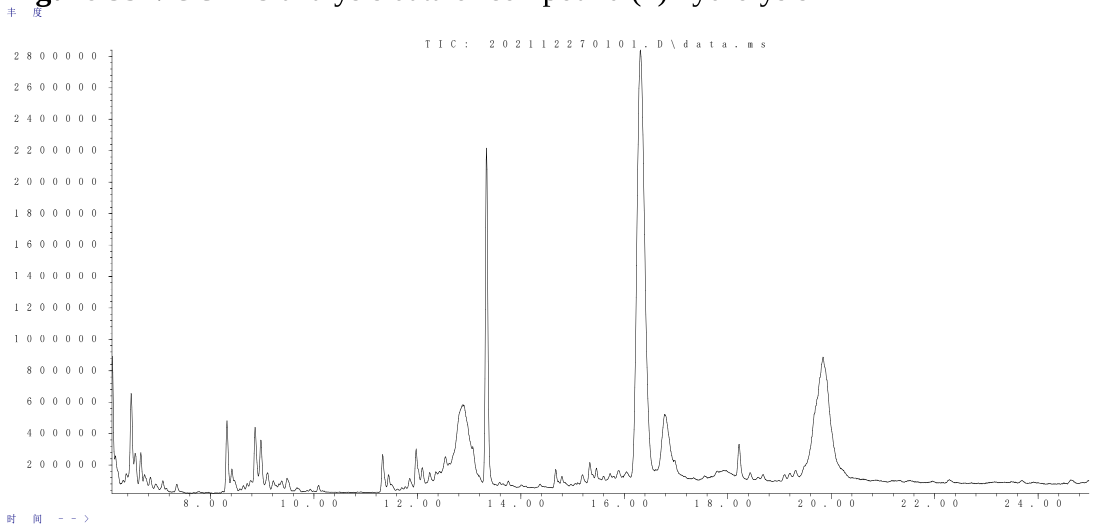

TIC of compound (**1**) hydrolysis

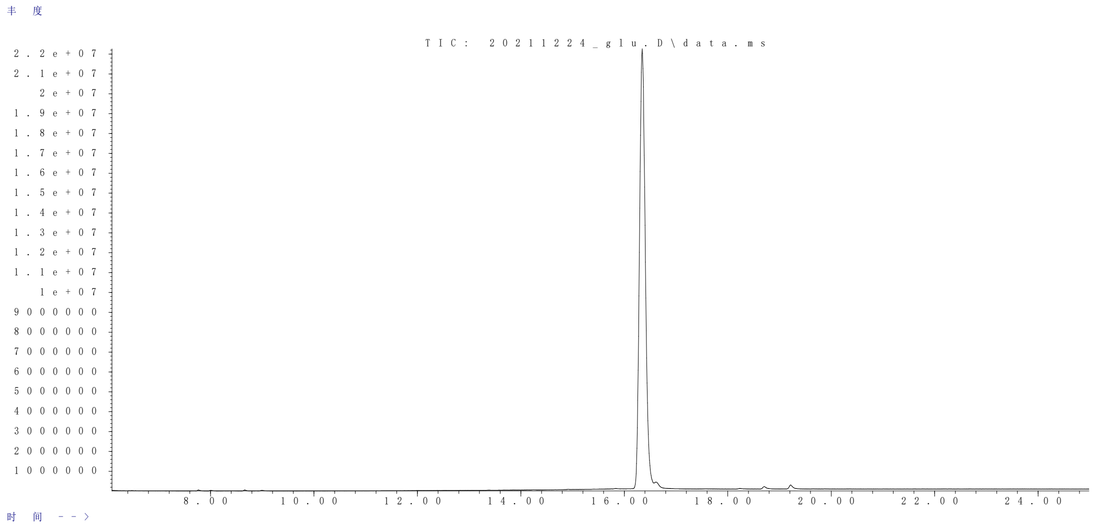

TIC of *D*-glucose standard samples

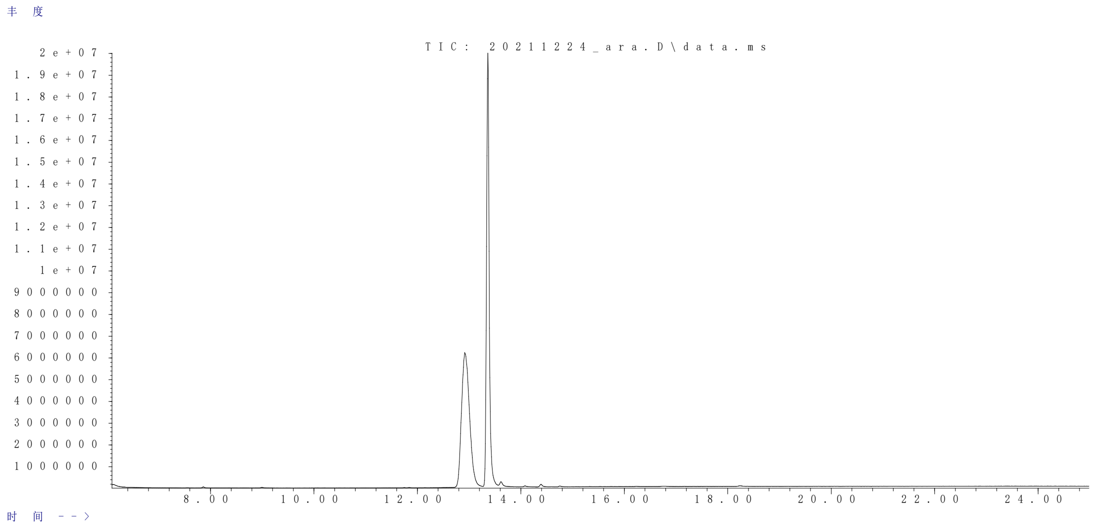

TIC of *L*-arabinose standard samples

Figure S52. GC-MS analysis data of compound (**2**) hydrolysis

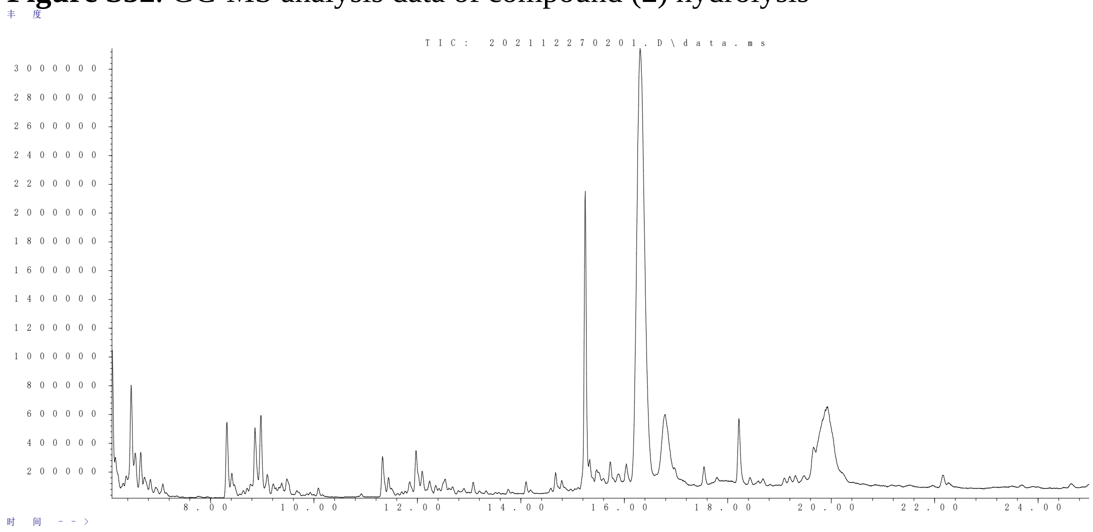

TIC of compound (**2**) hydrolysis

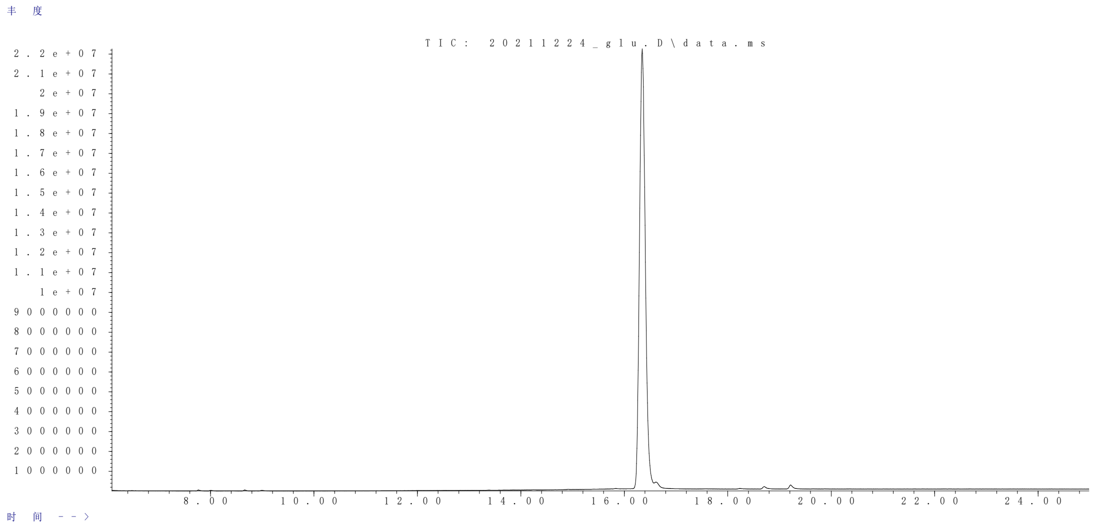

TIC of *D*-glucose standard samples

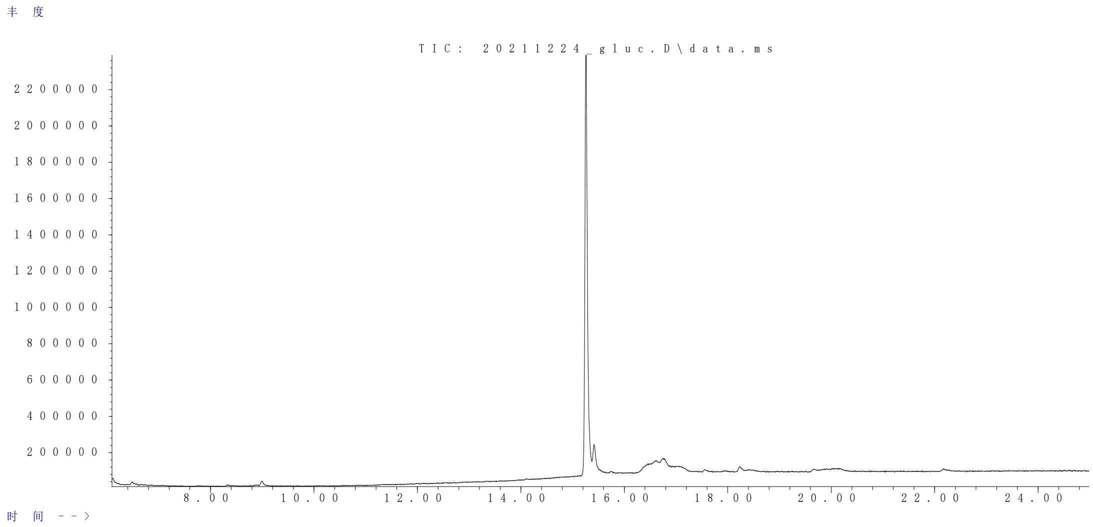

TIC of *D*-glucuronic acid standard samples

Figure S53. GC-MS analysis data of compound (**3**) hydrolysis

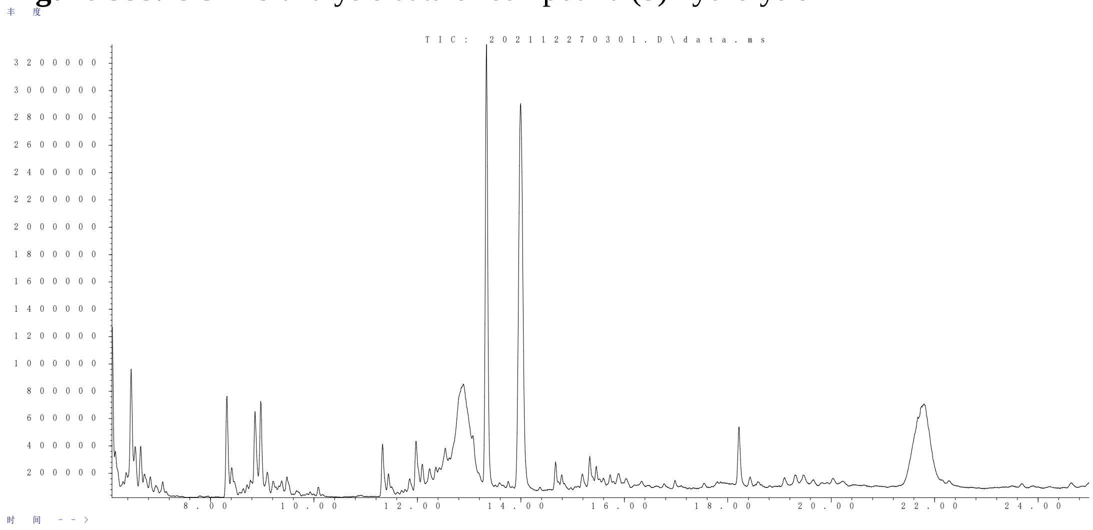

TIC of compound (**3**) hydrolysis

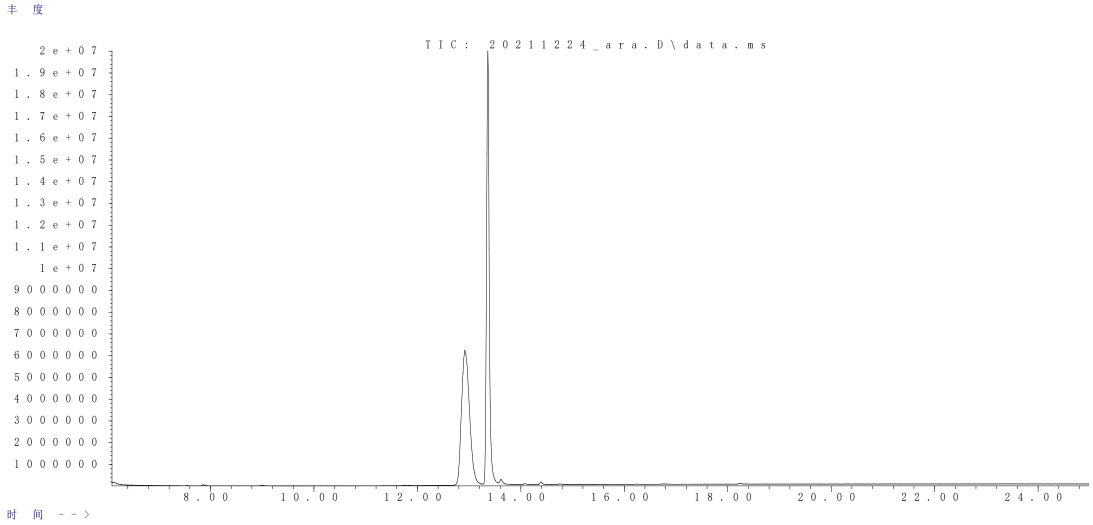

TIC of *L*-arabinose standard samples

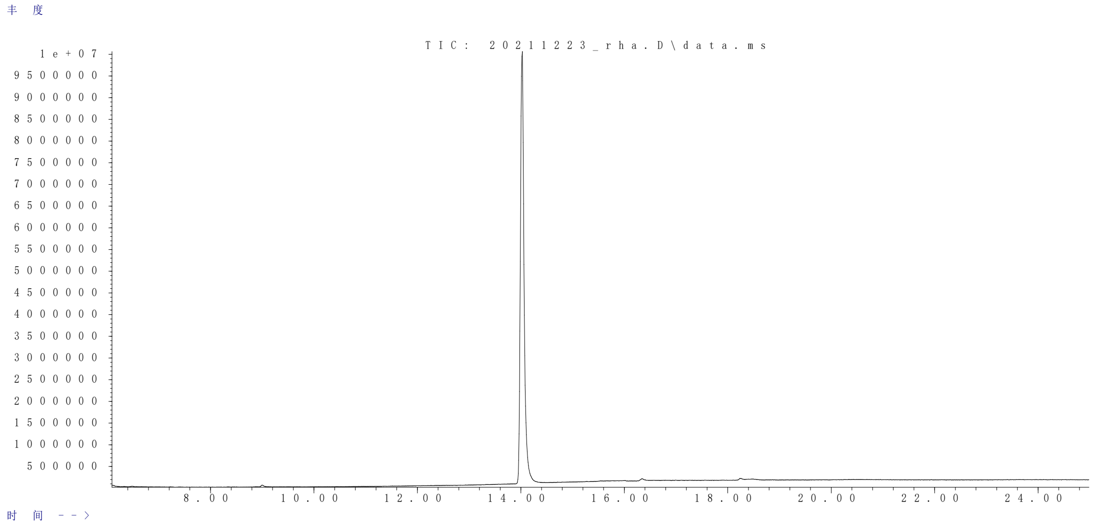

TIC of *L*-rhamnose standard samples

Figure S54. GC-MS analysis data of compound (**4**) hydrolysis

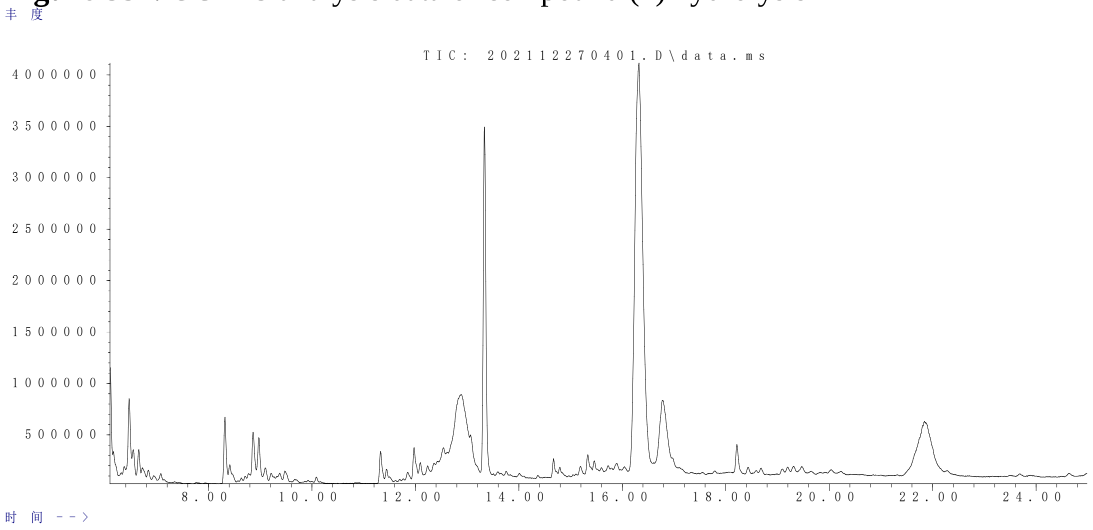

TIC of compound (**4**) hydrolysis

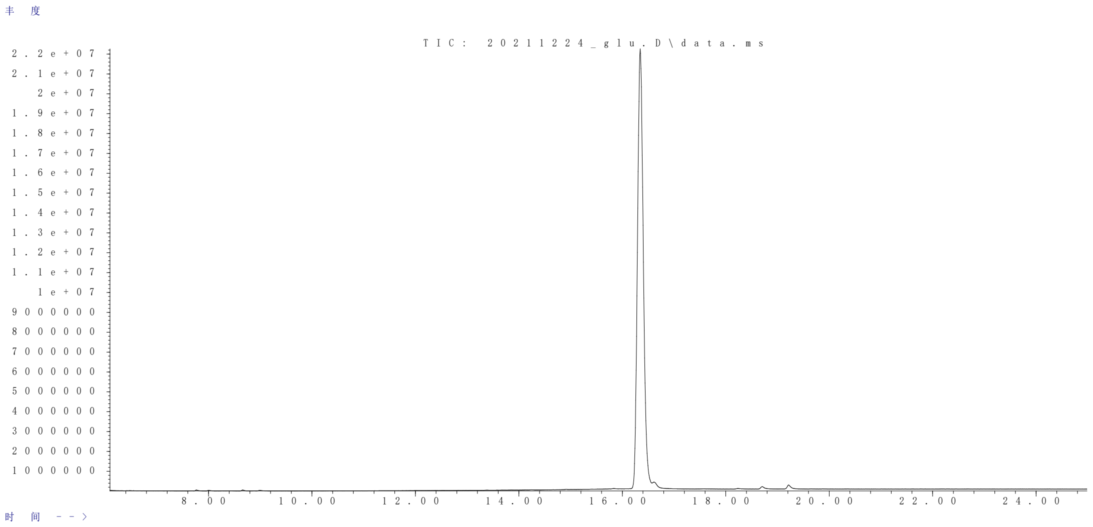

TIC of *D*-glucose standard samples

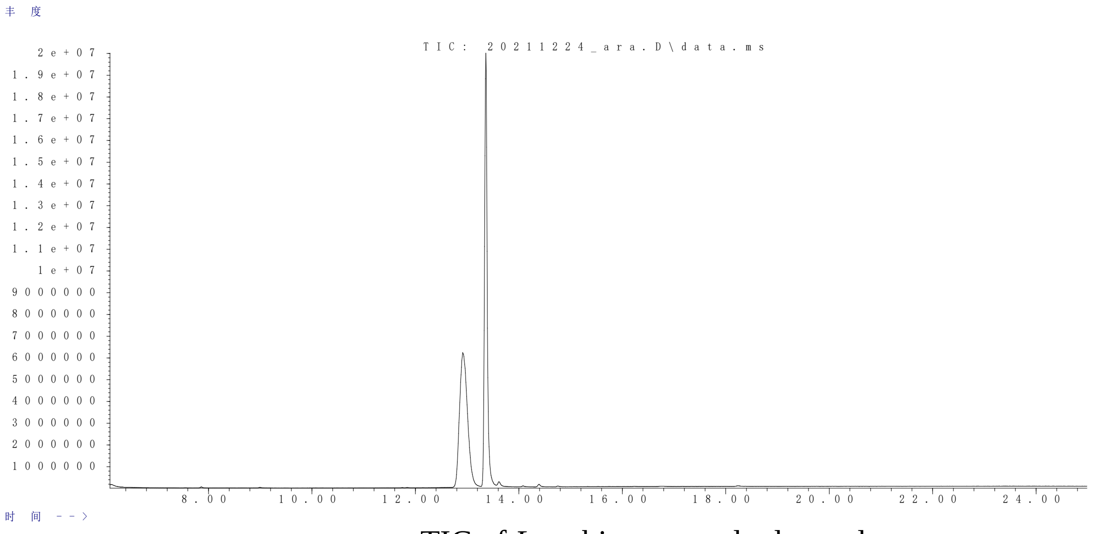

TIC of *L*-arabinose standard samples

Figure S55. GC-MS analysis data of compound (**5**) hydrolysis

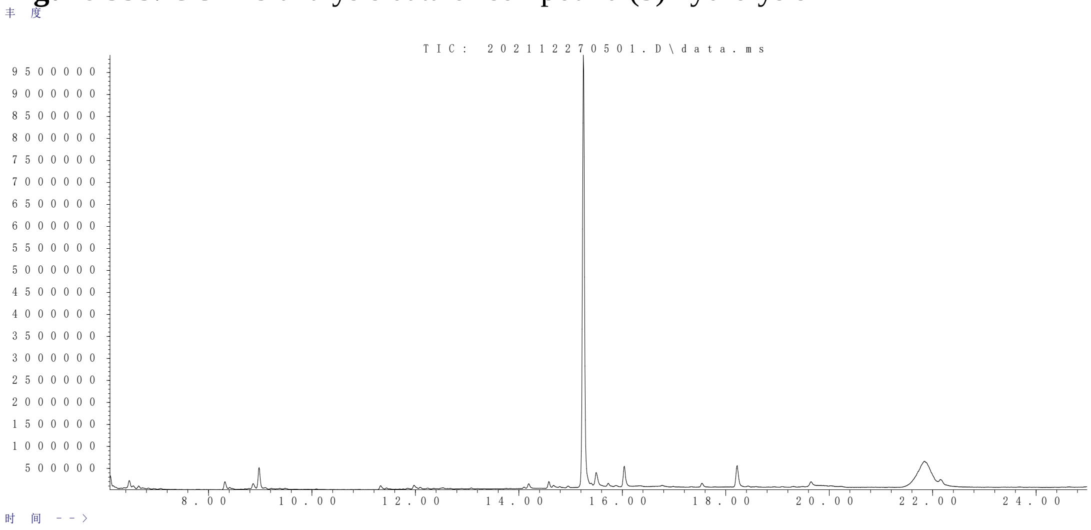

TIC of compound (**5**) hydrolysis

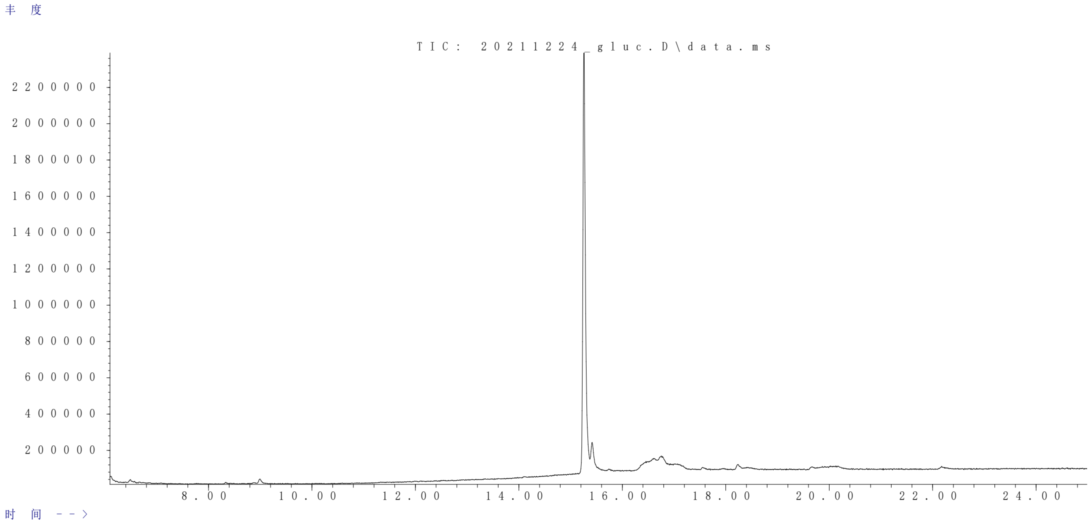

TIC of *D*-glucuronic acid standard samples

Figure S56. Structures of compounds **6-29**

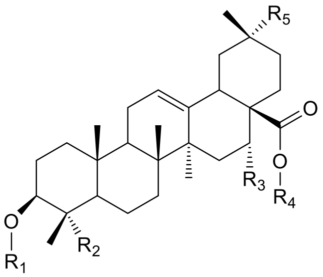

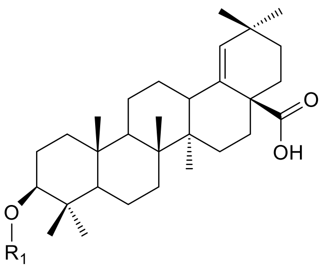

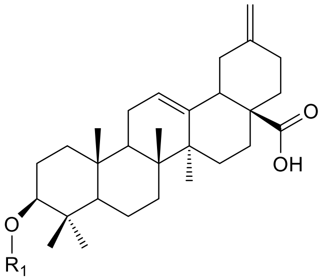

	R_1	R_2	R_3	R_4	R_5
**6**	Glc	CH_2OH	H	H	CH_3
**7**	Ara	CH_2OH	H	H	CH_3
**8**	GlcA	CH_3	H	H	CH_3
**9**	GlcA	CH_2OH	H	H	CH_3
**10**	GlcA	CH_3	OH	H	CH_3
**11**	GlcA	CHO	H	H	CH_3
**12**	GlcA	CH_2OH	OH	H	CH_3
**13**	6'-O-Me-GlcA	CH_2OH	H	H	CH_3
**14**	6'-O-Me-GlcA	CH_3	OH	H	CH_3
**15**	6'-O-Me-GlcA	CHO	H	H	CH_3
**16**	6'-O-Me-GlcA	CH_3	H	H	CH_3
**17**	-Ara2-Rha	CH_3	H	H	CH_2OH
**18**	-Ara2-Rha	CH_3	H	H	CH_3
**19**	Ara	CH_2OH	H	Glc	CH_3
**20**	-Ara2-Glc	CH_3	H	H	CH_3
**21**	-Ara3-Glc	CH_3	H	H	CH_3
**22**	Glc	CHO	H	Glc	CH_3
**23**	Glc	CH_3	H	Glc	CH_3
**24**	Glc2-Glc	CH_3	H	H	CH_3
**25**	6'-O-Me-GlcA	CH_2OH	H	Glc	CH_3
**26**	-Ara2-Rha	CH_3	H	Glc	CH_3

	R_1
**27**	-Ara2-Glc

	R_1
**28**	-Ara2-Glc
**29**	-Ara3-Glc

Table S1. ^{13}C-NMR (600MHz, in pyridine-*d*5) of compounds **6-29** (*δ* in ppm).

**No.**	**6**	**7**	**8**	**9**	**10**	**11**	**12**	**13**	**14**	**15**	**16**	**17**
**1**	38.6	38.7	38.6	38.5	38.6	38.0	38.6	38.5	38.6	37.9	38.6	38.7
**2**	25.8	26.1	26.6	25.8	26.6	25.0	25.9	26.0	26.6	25.1	26.6	26.4
**3**	82.1	81.8	89.0	81.9	89.0	81.9	82.0	82.1	89.1	82.1	89.1	88.6
**4**	43.4	43.4	39.5	43.3	39.5	55.4	43.4	43.4	39.5	55.4	39.5	39.4
**5**	47.6	47.6	55.7	47.4	55.8	47.6	47.2	47.4	55.8	47.8	55.7	55.8
**6**	18.1	18.1	18.4	18.0	18.4	20.4	18.2	18.0	18.4	20.3	18.4	18.4
**7**	32.8	32.8	33.2	32.8	33.4	32.4	33.2	32.7	33.4	33.2	33.2	33.0
**8**	39.7	39.7	39.7	39.6	39.8	39.9	39.9	39.6	39.8	39.9	39.7	39.6
**9**	48.0	48.1	48.0	48.0	47.1	47.8	47.6	48.0	47.1	47.7	48.0	47.9
**10**	36.9	36.9	37.0	36.8	37.0	36.0	36.9	36.8	37.0	36.0	36.9	36.9
**11**	23.7	23.6	23.7	23.5	23.7	23.6	23.8	23.5	23.7	23.7	23.7	23.7
**12**	122.5	122.5	122.5	122.4	122.4	122.2	122.4	122.4	122.3	122.1	122.5	122.4
**13**	144.8	144.8	144.8	144.7	145.1	144.8	145.1	144.7	145.1	144.8	144.8	144.9
**14**	42.1	42.1	42.1	42.0	42.1	42.1	42.0	41.8	42.1	42.1	42.1	42.0

**15**	28.2	28.3	28.2	28.2	36.2	28.2	36.2	28.2	36.1	28.2	28.3	28.2
**16**	23.8	23.7	23.8	23.6	74.7	23.7	74.7	23.6	74.7	23.6	23.7	23.6
**17**	46.6	46.6	46.6	46.5	48.8	46.4	48.8	46.3	48.8	46.6	46.6	47.0
**18**	41.9	41.9	42.0	41.8	41.4	41.9	41.3	42.0	41.4	41.9	42.0	41.1
**19**	46.3	46.3	46.4	46.2	47.2	46.6	47.2	46.5	47.2	46.4	46.4	41.3
**20**	30.8	30.9	31.0	30.8	31.0	30.9	31.0	30.8	31.0	30.9	31.0	36.5
**21**	34.1	34.1	34.2	34.1	36.1	34.2	36.2	34.1	36.2	34.1	34.2	29.0
**22**	33.1	33.2	33.3	33.1	32.9	33.1	32.8	33.1	32.8	32.4	33.2	32.6
**23**	64.7	64.4	28.3	64.3	28.1	206.8	64.5	64.2	28.1	206.7	28.2	28.0
**24**	13.6	13.6	17.0	13.5	16.9	10.3	13.6	13.5	16.9	10.3	16.9	16.9
**25**	16.0	16.0	15.4	15.9	15.5	15.5	16.2	15.9	15.5	15.4	15.4	15.4
**26**	17.4	17.4	17.4	17.3	17.4	17.3	17.5	17.3	17.4	17.2	17.4	17.3
**27**	26.1	26.1	26.2	26.1	27.2	26.1	27.2	25.9	27.2	26.1	26.2	26.1
**28**	180.1	180.1	180.2	180.0	179.9	180.1	179.9	180.1	180.0	180.1	180.2	180.2
**29**	33.2	33.1	33.2	33.1	33.3	33.2	33.3	33.1	33.3	33.1	33.3	73.8
**30**	23.6	23.8	23.7	23.7	24.7	23.6	24.7	23.7	24.7	23.6	23.8	19.7
**1'**	105.8	106.7	107.3	106.1	107.2	105.0	106.2	106.3	107.2	105.3	107.3	104.8
**2'**	75.8	73.1	75.5	75.1	75.5	75.0	75.0	75.3	75.4	77.7	75.4	75.8
**3'**	78.6	74.7	78.1	77.4	78.2	78.0	78.2	77.7	77.9	77.2	77.9	73.8
**4'**	71.5	69.6	73.4	73.3	73.5	73.3	73.5	73.1	73.2	74.9	73.2	69.8
**5'**	78.3	67.0	77.9	78.0	77.7	76.4	77.2	77.1	77.2	73.0	77.2	64.6
**6'**	62.7	-	172.8	173.4	173.2	173.4	172.6	170.7	170.8	170.6	170.8	-
**7'**	-	-	-	-	-	-	-	51.8	52.0	52.0	52.0	-
**1''**	-	-	-	-	-	-	-	-	-	-	-	101.6
**2''**	-	-	-	-	-	-	-	-	-	-	-	72.4
**3''**	-	-	-	-	-	-	-	-	-	-	-	72.3
**4''**	-	-	-	-	-	-	-	-	-	-	-	73.9
**5''**	-	-	-	-	-	-	-	-	-	-	-	68.6
**6''**	-	-	-	-	-	-	-	-	-	-	-	18.5

**No.**	**18**	**19**	**20**	**21**	**22**	**23**	**24**	**25**	**26**	**27**	**28**	**29**
**1**	38.8	38.8	38.6	38.7	38.0	38.6	38.6	38.6	38.8	39.0	38.6	38.7
**2**	26.5	26.0	26.4	26.6	25.0	26.0	26.5	25.9	26.4	26.6	26.4	26.6
**3**	88.7	81.8	88.7	88.6	81.6	88.8	88.9	82.1	88.6	88.7	88.7	88.6
**4**	39.4	43.4	39.4	39.7	55.4	39.4	39.6	43.4	39.4	39.6	39.4	39.6
**5**	55.9	47.6	55.7	55.8	47.5	55.7	55.7	47.4	55.8	55.9	55.7	55.8
**6**	18.5	18.1	18.4	18.4	20.4	18.4	18.4	18.0	18.4	18.3	18.4	18.4
**7**	33.2	33.0	33.2	33.2	32.4	32.4	33.2	32.4	33.0	34.9	33.1	33.1
**8**	39.7	39.9	39.6	39.6	40.1	39.8	39.4	39.8	39.8	40.9	39.6	39.7
**9**	48.0	48.1	47.9	48.0	47.8	47.9	47.9	48.0	48.0	51.3	47.9	47.9
**10**	37.0	36.9	36.9	37.0	36.0	36.9	36.9	36.8	36.9	37.0	36.9	36.9
**11**	23.8	23.8	23.7	23.7	23.2	23.6	23.7	23.3	23.5	21.2	23.7	23.7

**12**	122.5	122.9	122.4	122.5	122.5	122.8	122.4	122.8	122.8	26.4	122.9	122.9
**13**	144.8	144.0	144.8	144.7	144.1	144.1	144.8	144.0	144.0	41.6	144.1	144.1
**14**	42.1	42.1	42.1	42.1	42.1	41.7	42.1	42.0	42.0	42.9	41.9	42.0
**15**	28.3	28.2	28.2	28.2	28.1	28.2	28.1	28.2	28.0	29.9	28.2	28.2
**16**	23.6	23.3	23.7	23.7	23.6	23.3	23.7	23.7	23.3	34.3	23.7	23.7
**17**	46.6	46.9	46.6	46.6	46.9	46.9	46.6	46.8	46.9	48.5	46.9	47.0
**18**	41.9	41.7	41.9	41.9	41.6	42.0	41.9	41.6	41.6	138.9	47.8	47.8
**19**	46.4	46.1	46.4	46.4	46.1	46.1	46.4	46.0	46.1	132.0	42.0	41.9
**20**	30.9	30.7	30.9	30.9	30.7	30.7	30.9	30.6	30.7	32.3	149.0	149.1
**21**	34.2	33.9	34.1	34.1	33.9	33.9	33.1	33.9	33.9	34.2	30.3	30.3
**22**	33.1	32.5	33.1	33.1	32.3	33.0	33.1	32.7	32.4	34.1	38.3	38.3
**23**	28.0	64.4	28.1	28.0	206.8	28.2	28.2	64.2	28.1	28.0	28.2	28.1
**24**	17.0	13.6	16.7	16.9	10.3	16.9	16.8	13.5	16.8	16.5	16.7	16.9
**25**	15.5	16.1	15.4	15.4	15.5	15.5	15.4	16.0	15.5	16.8	15.4	15.4
**26**	17.3	17.5	17.3	17.3	17.3	17.4	17.3	17.4	17.4	16.2	17.2	17.3
**27**	26.1	26.1	26.1	26.1	26.0	26.5	26.1	26.0	26.0	15.2	26.0	26.1
**28**	180.1	176.4	180.1	180.1	176.3	176.4	180.1	176.3	176.4	179.0	179.3	179.4
**29**	33.2	32.8	33.1	33.1	33.0	33.2	34.1	33.0	33.0	30.7	107.0	107.0
**30**	23.7	23.6	23.6	23.6	23.6	23.7	23.6	23.5	23.7	29.3	-	-
**1'**	104.9	106.7	104.8	107.3	104.7	106.8	105.0	106.3	104.7	104.8	104.8	107.4
**2'**	75.9	73.1	81.0	71.5	75.3	75.7	83.4	75.3	75.8	81.0	81.0	71.9
**3'**	73.9	74.7	73.4	84.1	78.6	78.7	78.3	77.1	73.9	73.4	73.4	84.1
**4'**	68.7	69.6	68.2	69.3	71.5	71.8	71.6	73.1	68.5	68.2	68.2	69.3
**5'**	64.8	67.0	64.9	67.0	78.5	78.2	77.9	77.7	64.5	64.9	64.9	67.0
**6'**	-	-	-	-	62.8	63.0	62.7	170.7	-	-	-	-
**7'**	-	-	-	-	-	-	-	51.9	-	-	-	-
**1"**	101.7	95.7	106.0	106.4	95.7	95.7	106.0	95.6	101.6	106.0	106.0	106.4
**2"**	72.4	74.1	76.4	75.7	74.0	74.0	77.1	74.0	72.3	76.4	76.4	75.7
**3"**	72.5	79.3	78.1	78.3	79.3	78.8	78.0	78.8	72.5	78.1	78.1	78.4
**4"**	74.0	71.0	71.5	71.9	71.0	71.0	71.5	71.0	74.0	71.5	71.5	71.5
**5"**	69.8	78.9	78.1	78.6	78.8	79.3	78.2	79.2	69.8	78.1	78.1	78.6
**6"**	18.5	62.1	62.5	62.6	62.1	62.1	62.6	62.1	18.4	62.5	62.5	62.6
**1'''**	-	-	-	-	-	-	-	-	95.6	-	-	-
**2'''**	-	-	-	-	-	-	-	-	73.6	-	-	-
**3'''**	-	-	-	-	-	-	-	-	78.8	-	-	-
**4'''**	-	-	-	-	-	-	-	-	71.0	-	-	-
**5'''**	-	-	-	-	-	-	-	-	79.2	-	-	-
**6'''**	-	-	-	-	-	-	-	-	62.1	-	-	-
